# Supplementary material for: Whole body regeneration deploys a rewired embryonic gene regulatory network logic
Source: Nat Commun. 2025 Dec 11;17:503. doi: 10.1038/s41467-025-67196-4 (PMC12804838; doi:10.1038/s41467-025-67196-4)
Supplement: Supplementary file 1 — Supplementary Information [file 41467_2025_67196_MOESM1_ESM.pdf]

Figure S1

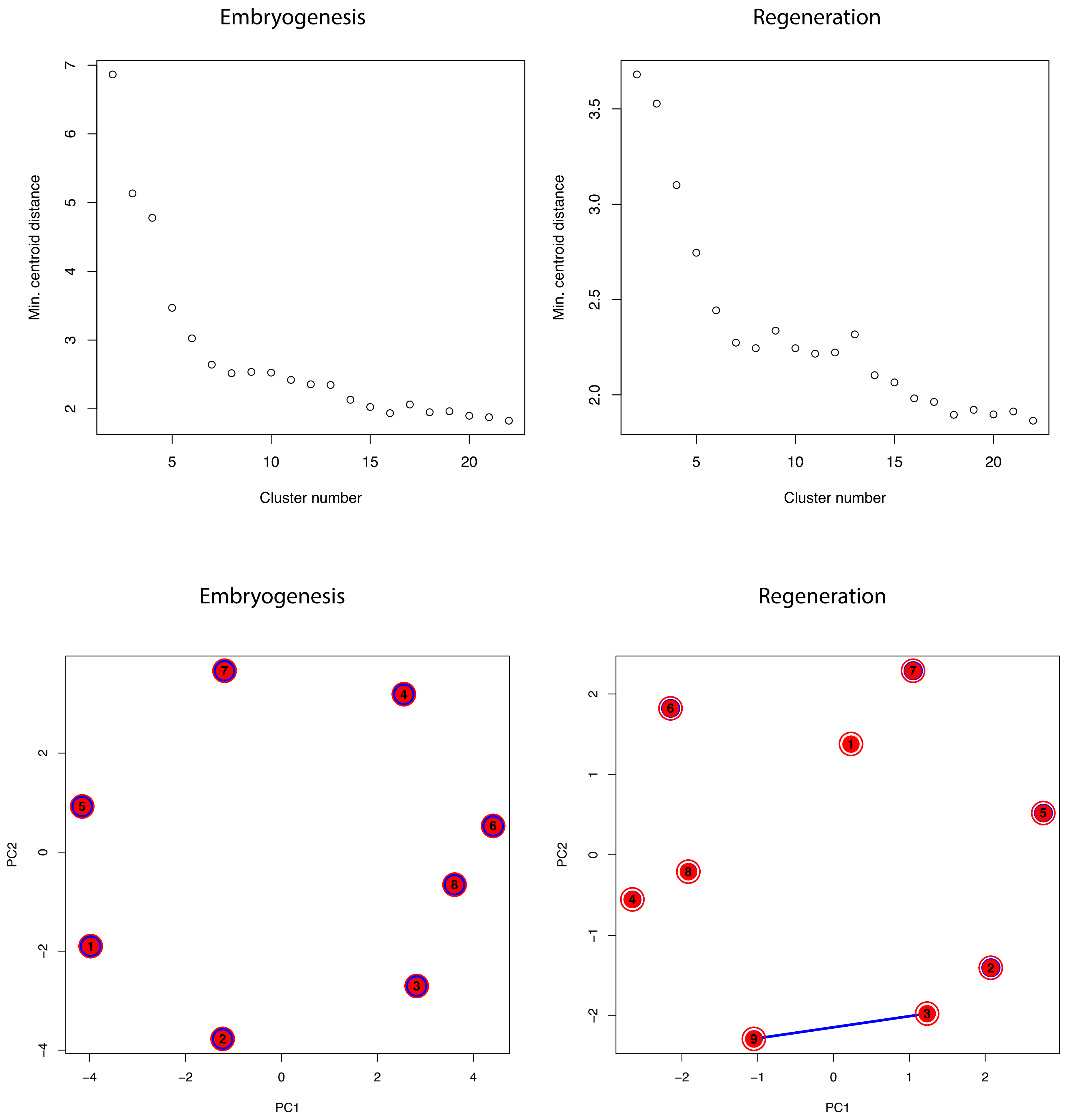

Figure S1: Fuzzy c-means clustering minimum centroid distance and cluster overlap. (Top): Minimum centroid distances for embryogenesis (left) and regeneration (right) clusters. (Bottom): Overlap plots for embryogenesis (left) and regeneration (right) clusters show principal component analysis of the cluster centers. The overlap is visualised by lines with variable width indicating the strength of the overlap.

Figure S2

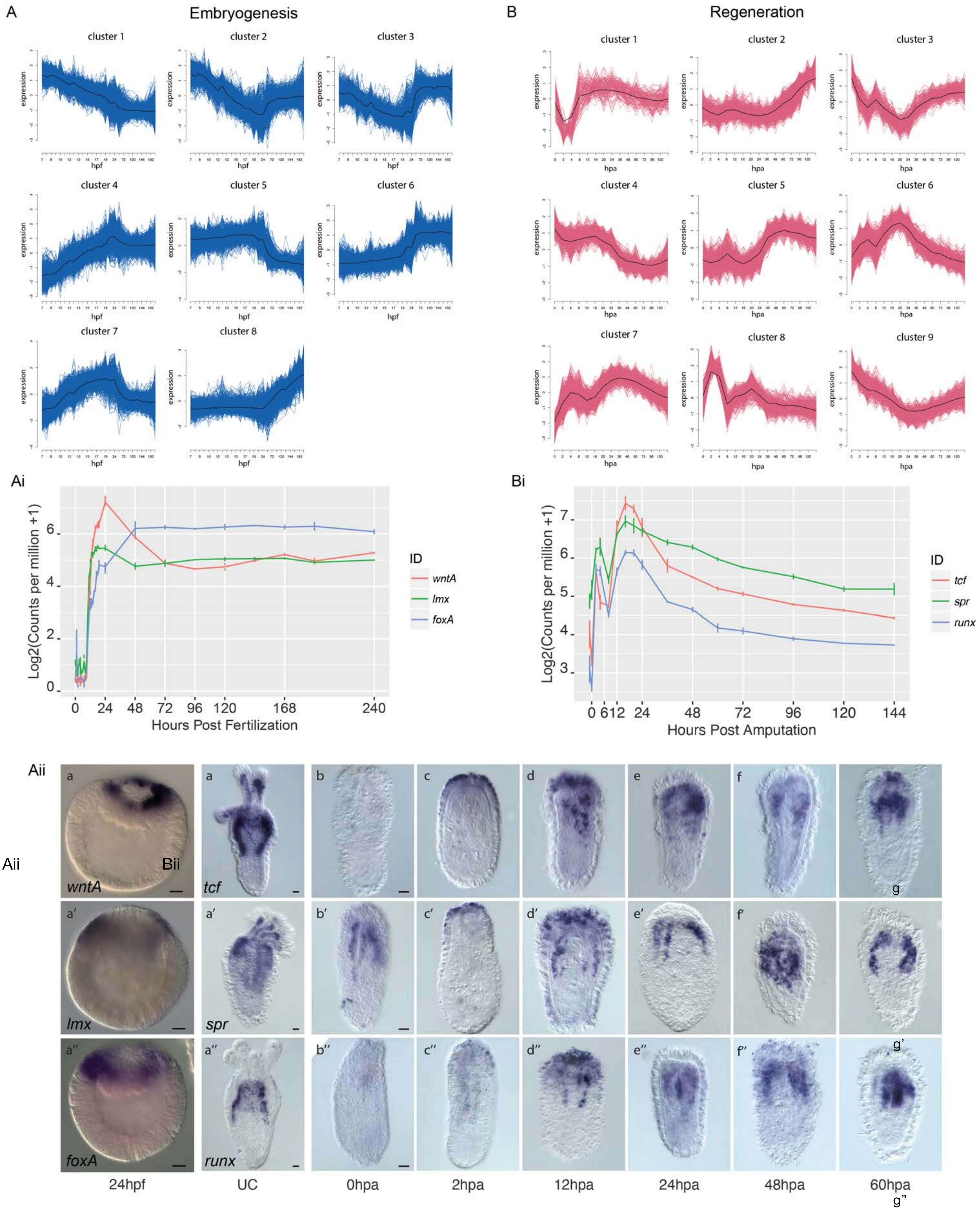

Figure S2: Embryonic and Regenerative gene expression forms discrete clusters. (A, B) Fuzzy c-means clustering of embryonic (A) and regeneration (B) gene expression. Each cluster is plotted with standardized expression along the y-axis and developmental time along the x-axis. Black trace denotes the cluster core (centroid). (Ai-Aii) Exemplar gene expression from a cluster activated early during embryogenesis in the cluster E-4. *wntA, lmx, foxA* are temporally co-expressed (Ai) and in situ hybridization at 24hpf confirms early activation of this gene cluster (Aii). (Bi-Bii) Exemplar gene expression from a cluster activated in the early regenerative program. *tcf, spr, runx* are all activated early in the cluster R6 and are temporally co-expressed (Bi) and *in situ* hybridization during regeneration confirms early activation and the expression dynamics of this gene cluster (Bii). Scale bar: 20µm

Figure S3

Down-regulated genes @20hpa

Reg cluster / gene dataset	total	R1	R2	R3	R4	R5	R6	R7	R8	R9
emb_MEK/ERK	6	/	1	3	1	1	/	/	/	/
emb_cWNT	4	/	/	2	2	/	/	/	/	/
regen_spe	44	/	/	13	3	/	/	/	/	28

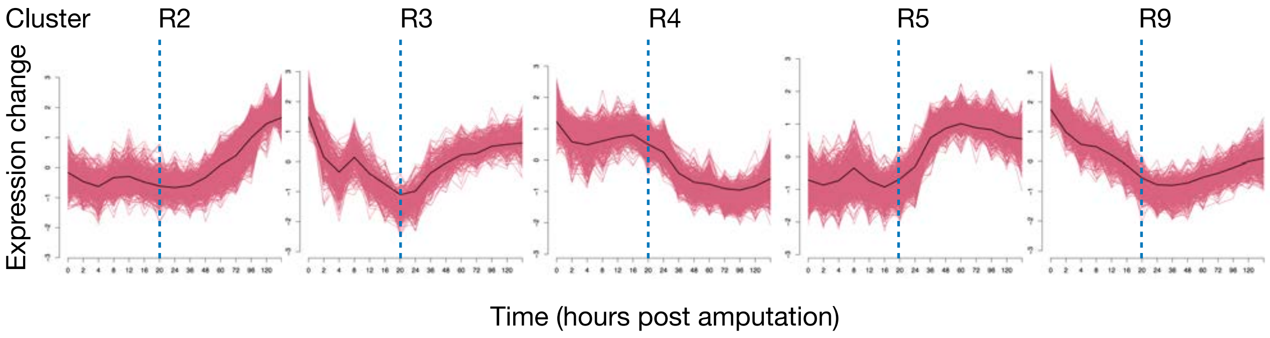

Figure S3: Overview of the down-regulated genes at 20hpa that belong to either of the three datasets (emb_MEK/ERK, emb_cWnt or regen_spe). Overview of the down-regulated genes at 20hpa that belong to either of the three datasets (emb_ERK, emb_WNT or regen_spe) highlighting the regeneration expression clusters they are assigned to. The same overview for the up-regulated genes can be found in Fig.S3B.

Figure S4

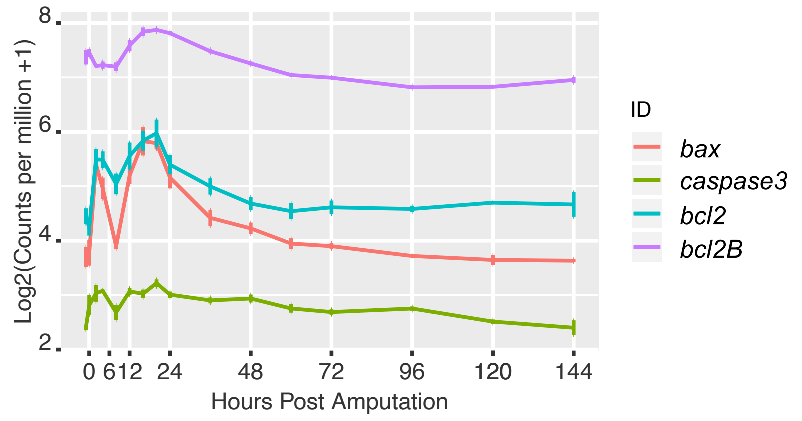

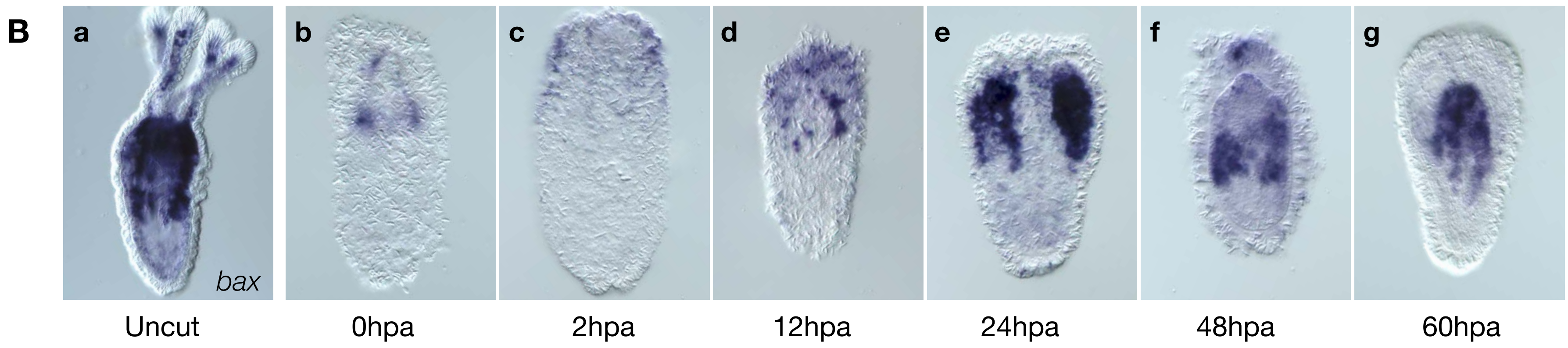

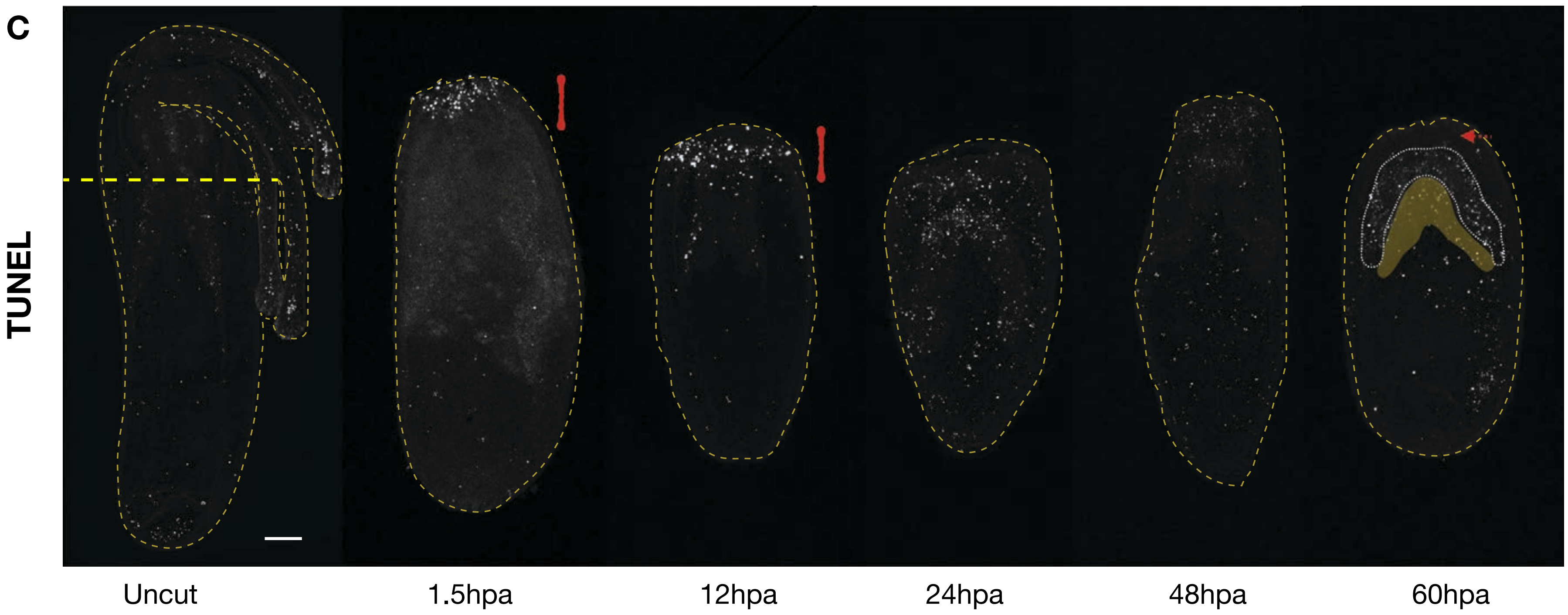

D

TUNEL+ cells	Uncut	1.5hpa	24hpa	48hpa	60hpa
% of TUNEL+ cells in oral part	13 %	66 %	38 %	35 %	69 %
TUNEL+ cells in oral part	11	93	170	79	98
TUNEL+ cells in whole body	83	140	462	226	144
number of replicate	5	5	5	5	5

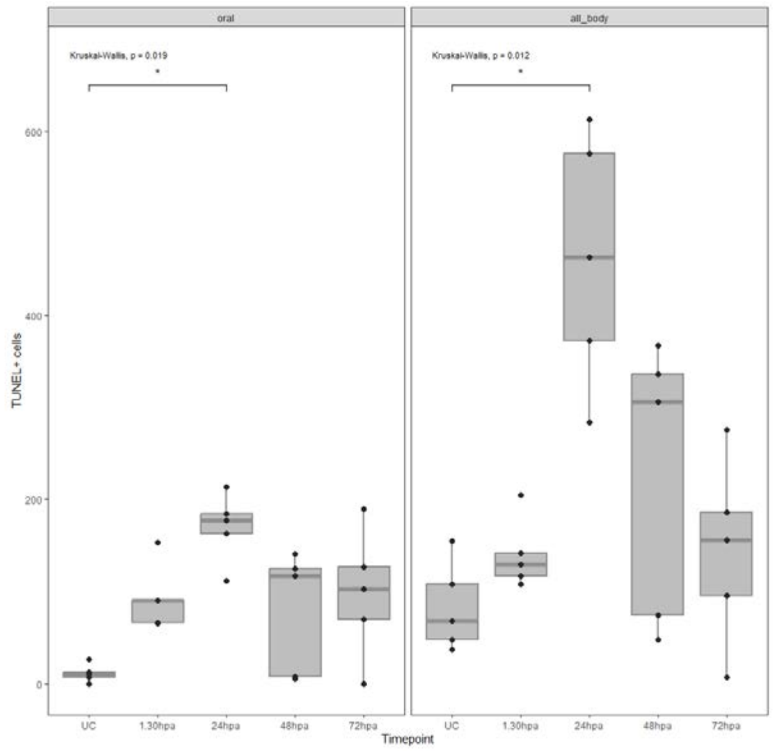

Figure S4: Additional information – Apoptosis. (A) Temporal expression patterns for *bax, caspase3, bcl2* and *bcl2b* (data obtained from NvERTx.ircan.org[39]. (B) Extended spatial *bax* expression pattern in uncut, 0, 2, 12, 24, 48 and 60hpa juvenile polyps. (C) TUNEL staining (white dots) in uncut controls and during regeneration (1.5hpa – 60hpa). The red bar highlights the amputation site with increased TUNEL+ cells and the yellow patch indicates the tissues that link the mesenteries to the endodermal layer of the body wall (patch define by the white discontinued line). The yellow dashed line indicates the sub-pharyngeal amputation plane, and the red arrow designates the amputation site epithelia devoid of TUNEL+ cells. (D) Cell count and ratio of TUNEL positive cells (TUNEL+) detected at the amputation site *vs* the entire body. *n* indicates the number of analyzed animals. The dot plots represent the distribution of the samples.

Figure S5

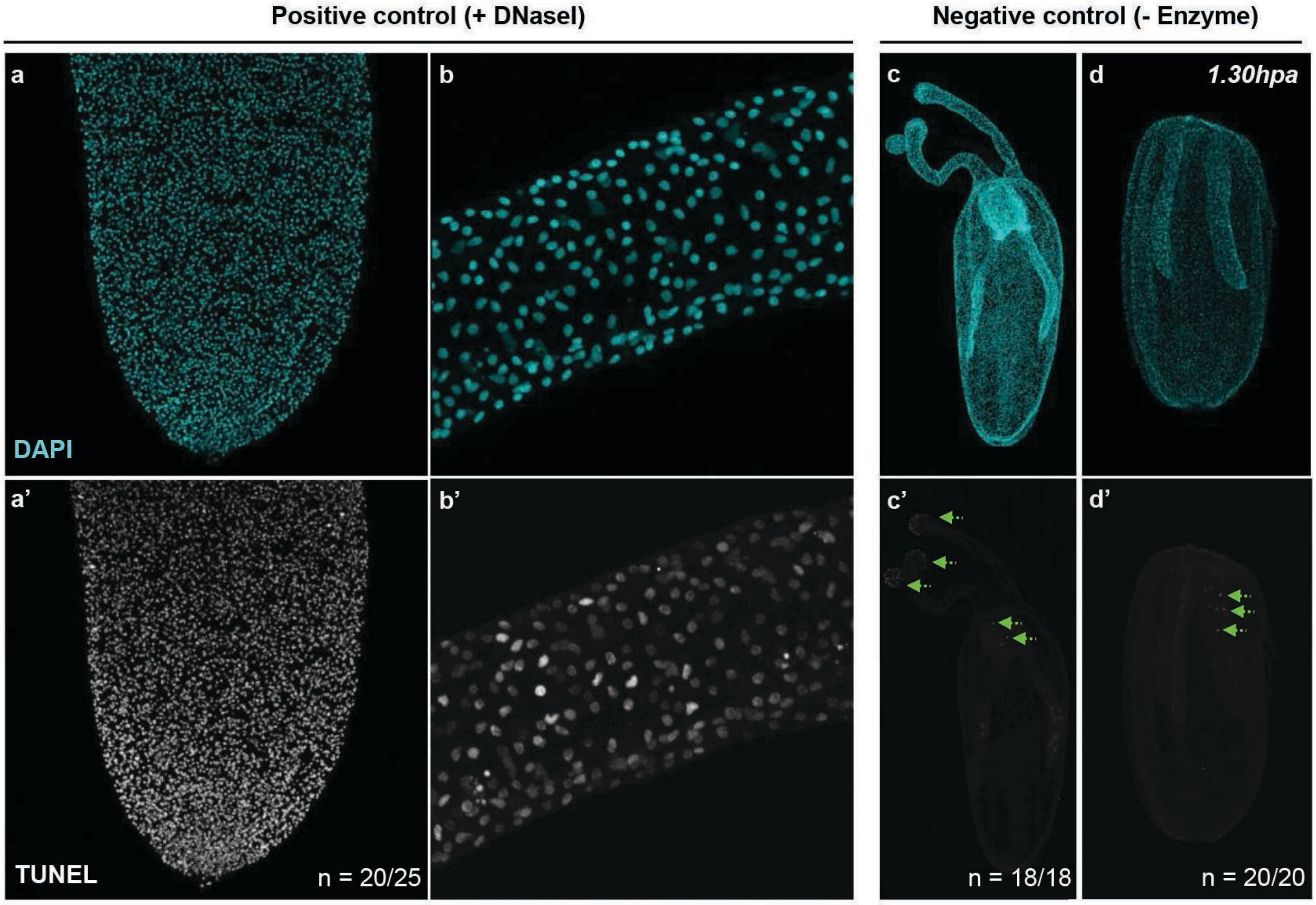

Figure S5: TUNEL assay controls. Close-up of the physa (a, a') and the tentacles (b, b') for the TUNEL assay positive control using DNAse I treatment. Uncut polyp (c, c') and 1.50hpa (d, d') for the TUNEL assay negative control without Enzyme. Green dashed arrows (b', c') indicate non-specific auto-fluorescent staining. n=[number of specimen with represented phenotype]/[total number of analyzed specimen]

Figure S6

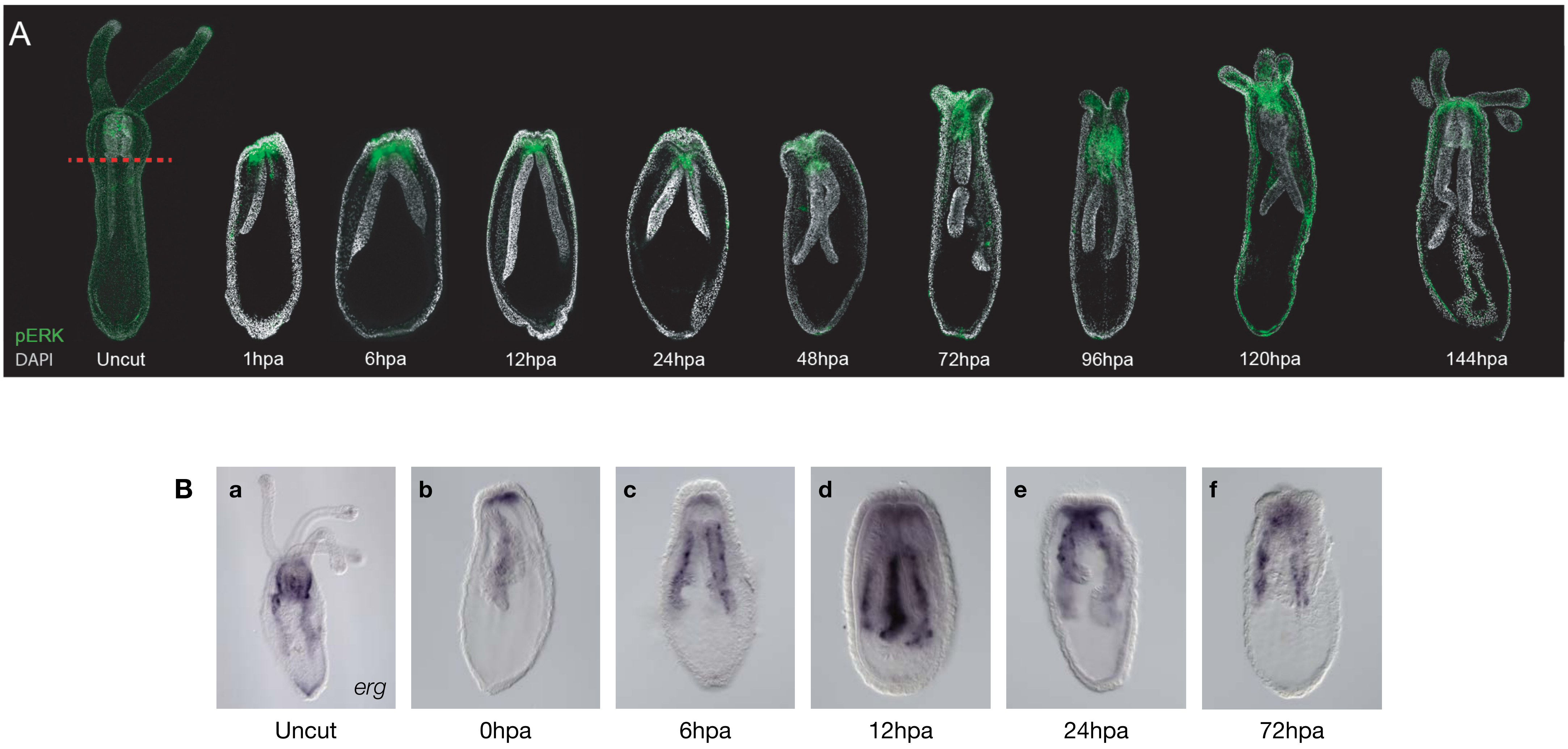

Figure S6: pERK is activated and localized at the amputation site throughout regeneration. (A) Complementary images of the Immunohistochemistry using an anti-pERK antibody revealing the activated/phosphorylated form of ERK (green) in uncut and regenerating polyps from 1 hpa to 72 hpa (counterstaining: DAPI). the red dashed line indicates the sub-pharyngeal amputation plane. (B) Extended spatial *erg* expression pattern in uncut, 0, 6, 12, 24 and 72hpa juvenile polyps.

Figure S7

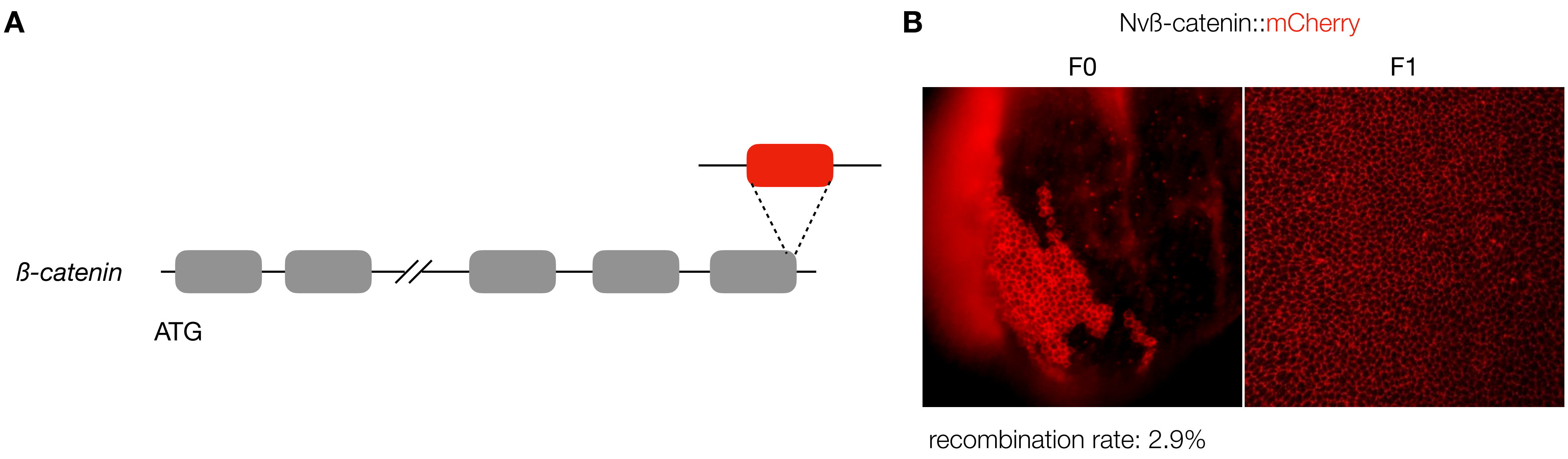

Figure S7: Establishment of a C-terminal mCherry fusion to ß-catenin; Nvß-catenin::mCherry. (A) Schematic illustration of the Nvß-catenin gene and site of C-terminal mCherry transgene fusion. (B) F0 polyp displaying mosaic expression (recombination rate 2.9%) and heterozygous F1 polyp (germline transmission). The red staining (mCherry expression) indicates the membrane localization of ß-catenin.

Figure S8

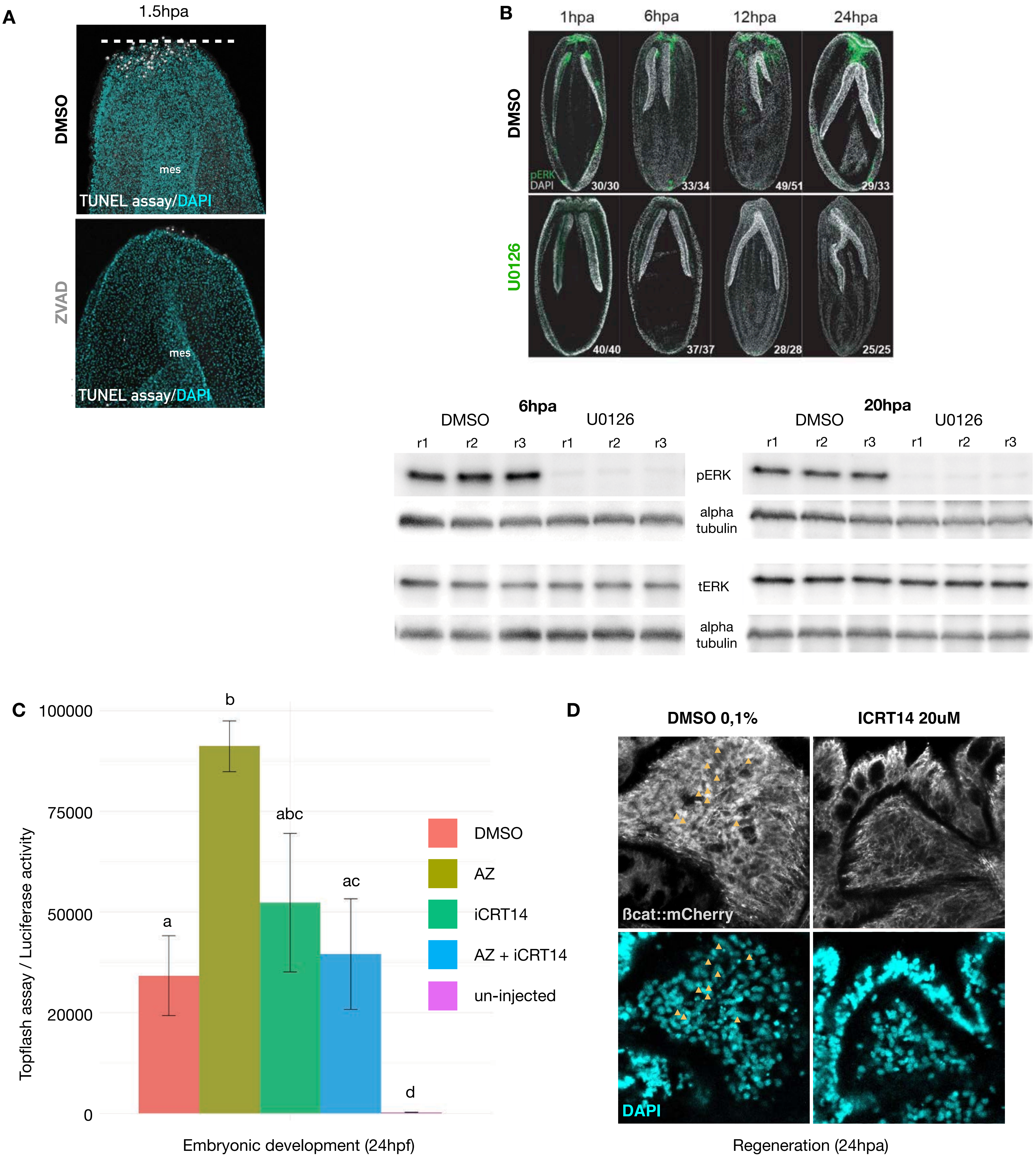

Figure S8: Z-VAD, U0126 and iCRT14 efficiently block apoptosis, pERK and cWnt. (A) Close-up of the amputation site in DMSO or Z-VAD treated polyps, fixed and stained with DAPI (turquoise) and TUNEL (white). (B) Immunohistochemistry and Western blot using an anti-pERK antibody (green) at various moments during regeneration in the absence or presence of the MEK inhibitor U0126 (Control condition: DMSO). In addition to anti-pERK, anti-tERK (total ERK) and anti-alpha-tubulin antibodies were used as controls for the western blot. The raw blots used for Fig. S8B can be found in the following poges. (C) Embryos injected with the TOP FLASH construct and treated with DMSO (yellow column), 1-Azakenapaullone (AZ - cWnt activator; orange column) or iCRT14 (cWnt inhibitor; red column). Different letters above the bars indicate statistically significant differences among treatments ($p < 0.05$) according to two-sided pairwise Kruskal–Wallis tests followed by post hoc Dunn tests with Benjamini–Hochberg adjustment. Statistical analyses are provided in Sup Table 26. (D) iCRT14 prevents nuclearization of ß-catenin during regeneration. mCherry IHC on regenerating (24hpa) polyps treated with DMSO or iCRT14. White arrowheads indicated nuclearized ß-catenin.

Figure S8 - original blots (pERK)

pERK

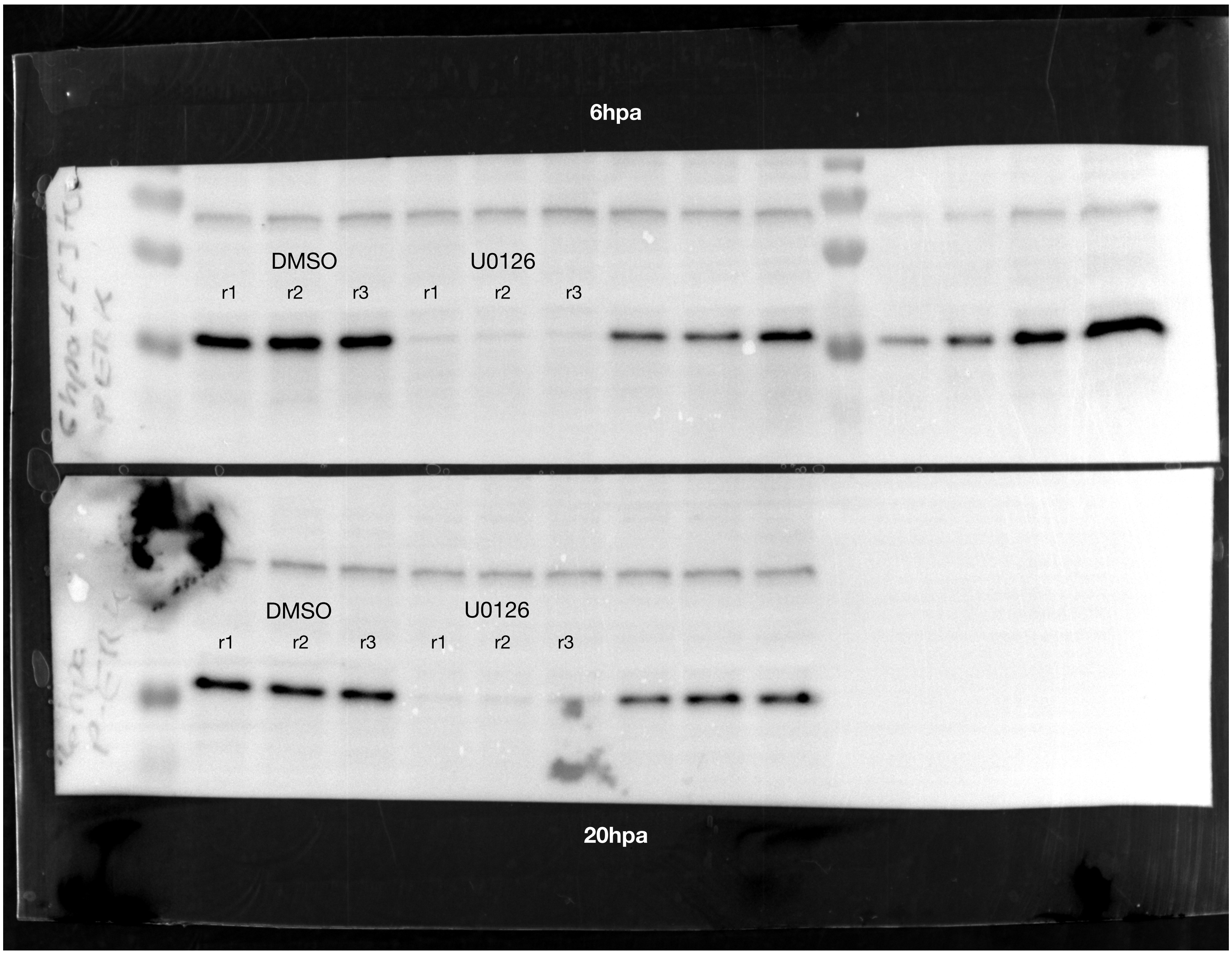

alpha-tubulin for pERK

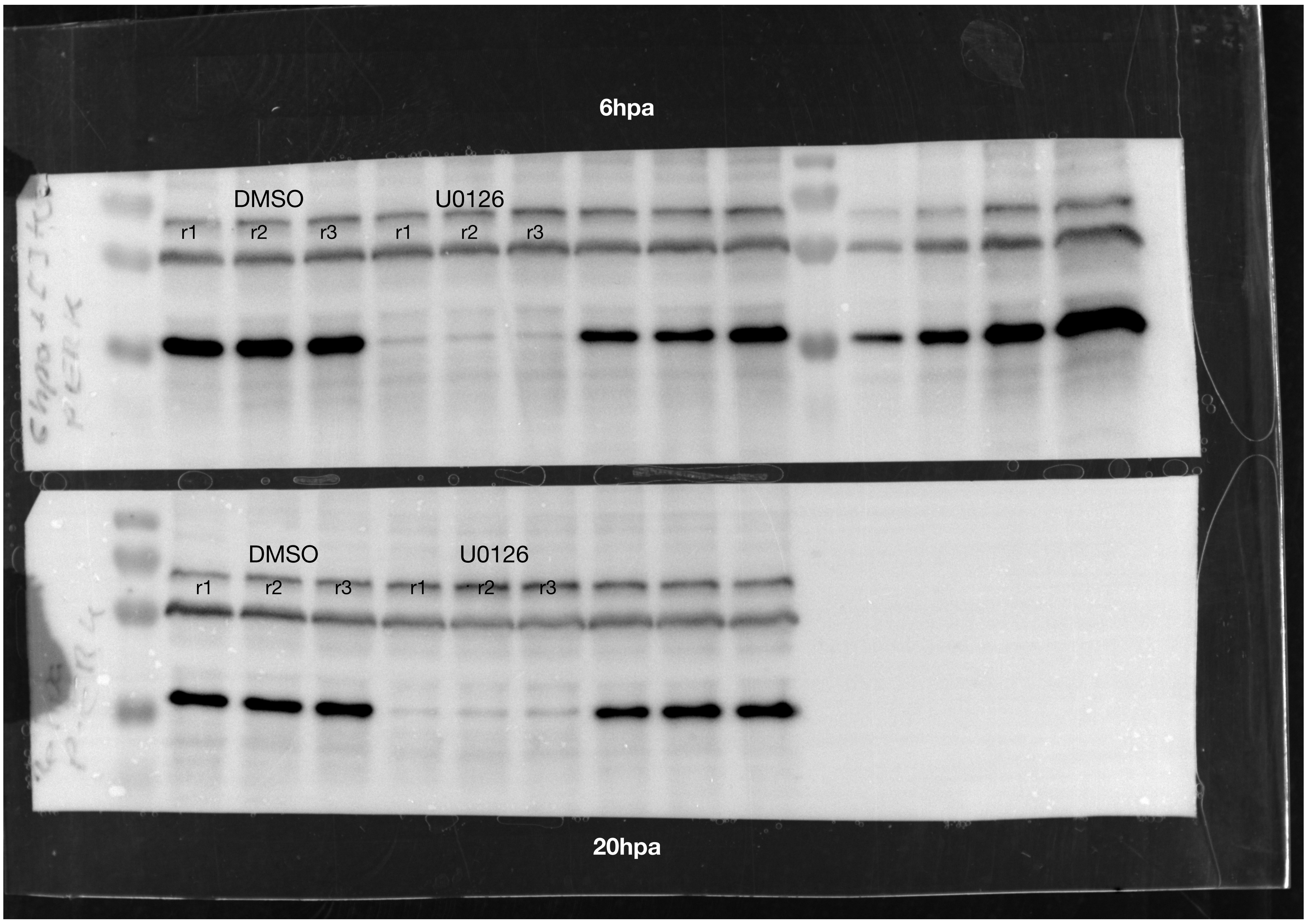

Figure S8 - original blots (tERK - ERK total)

tERK

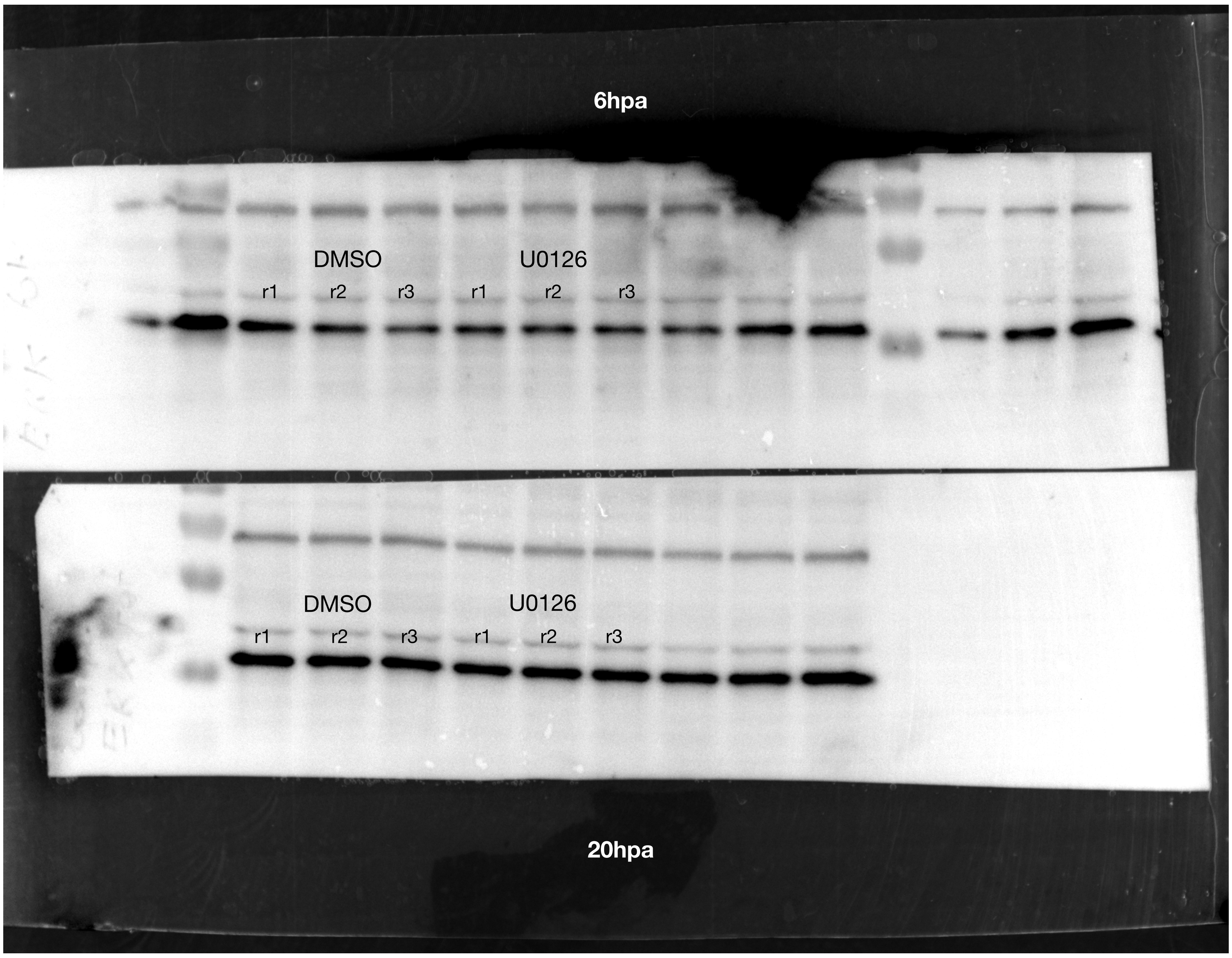

alpha-tubulin for tERK

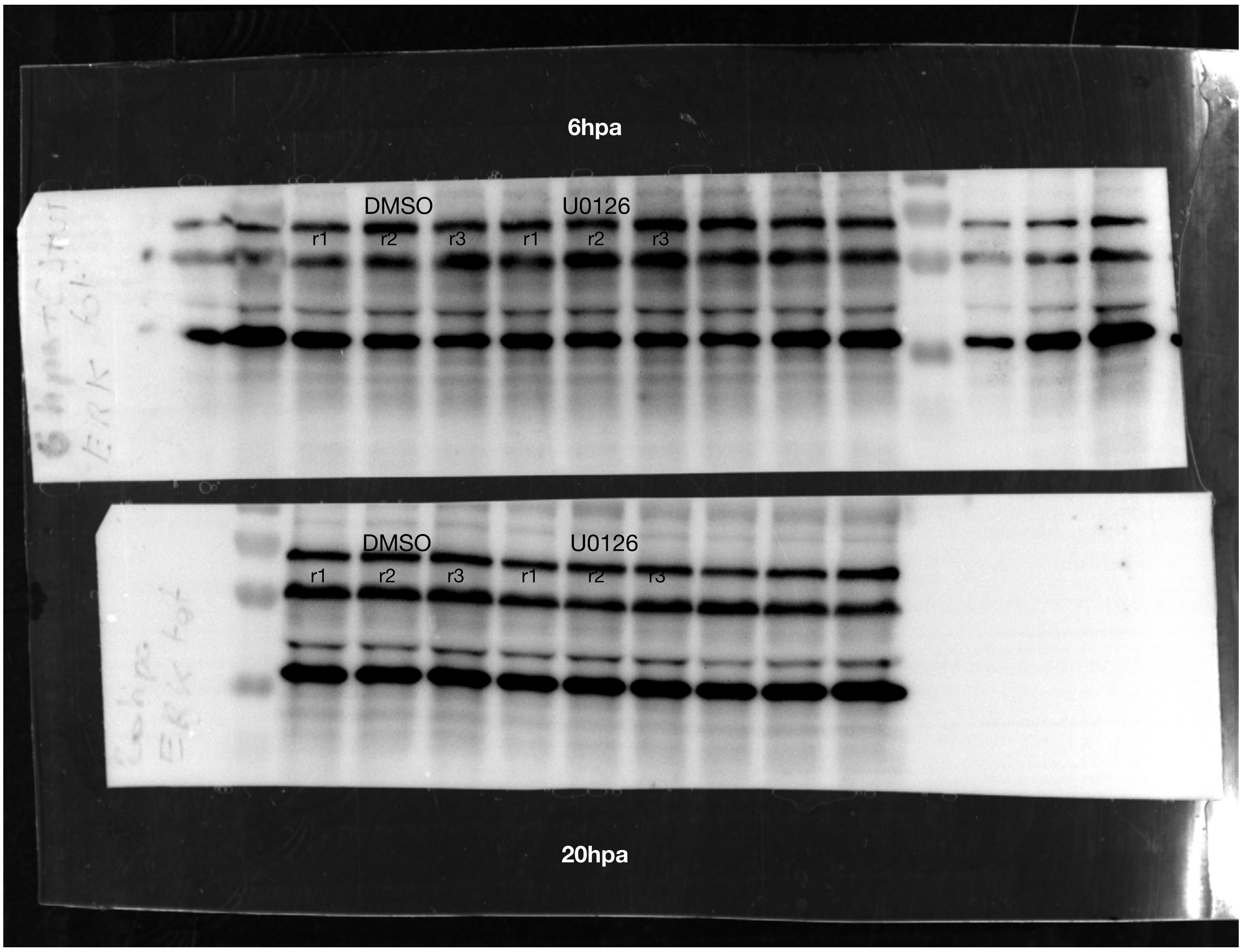

Figure S9

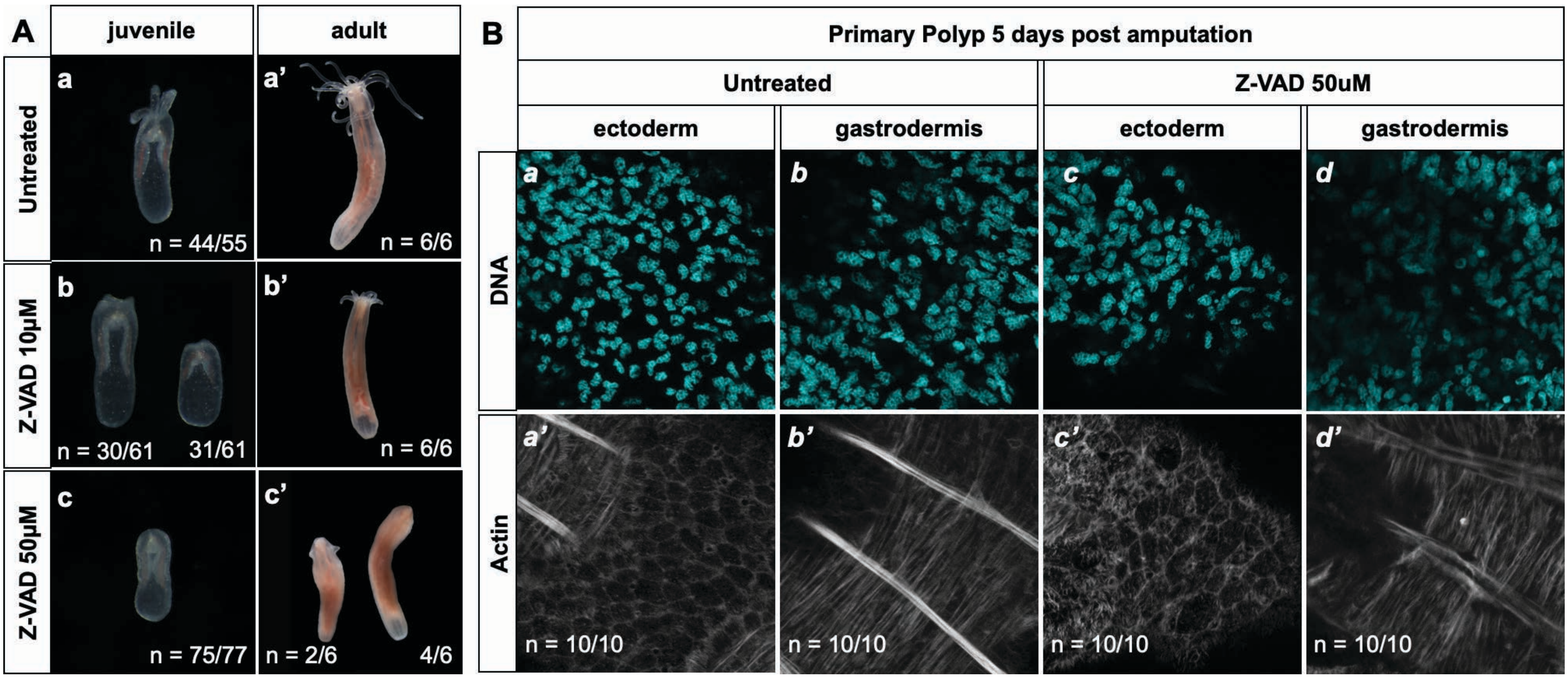

Figure S9: Z-VAD dose-response assay and effects on tissue organization. (A) Z-VAD dose-response assay on juvenile (a, b, c) vs adult (a', b', c') amputated polyps. Untreated polyps at 5 days post amputation (5dpa) (a, a'), 10µm Z-VAD treated polyp at 5dpa (b, b') and 50µm Z-VAD treated polyp at 5dpa (c, c'). 50µm Z-VAD treatment induces the inhibition of regeneration in nearly 100% of the total number of cases (75 out of 77 polyps, c), while 10µm Z-VAD treatment 743 fully inhibits regeneration in only half of the total number of cases (31 out of 61 polyps, b). (B) Z-VAD treatment does not affect tissues organization. Untreated tissues (a, a', b, b') and 50µm Z-VAD treated tissues (c, c', d, d') at 5dpa. Nucleus DNA staining (DAPI) is in blue (a,b,c,d). Cell membrane and muscle network actin filaments staining (phalloidin) are in white (a',b',c',d').

Figure S10

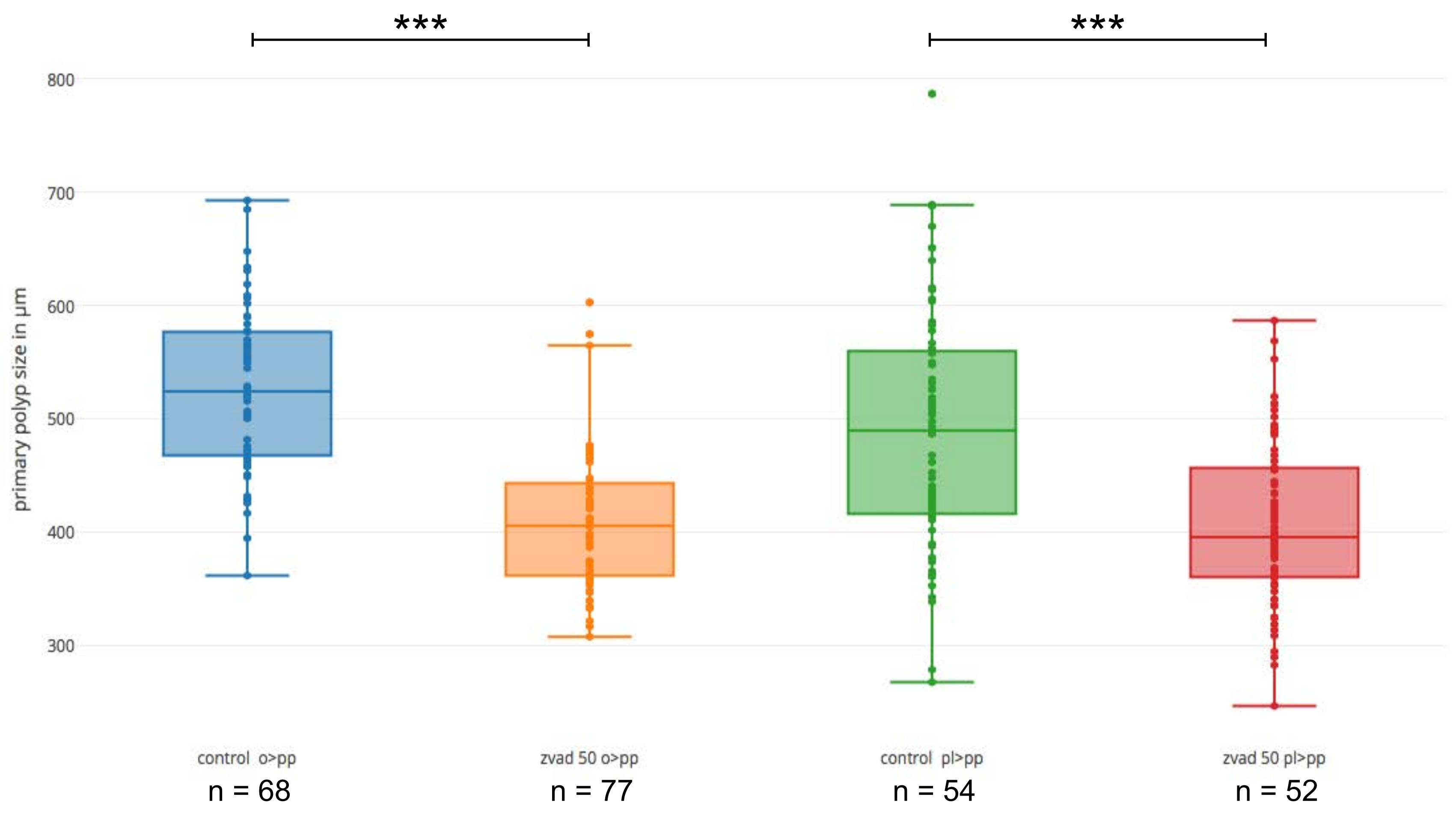

Figure S10: Apoptosis seems required for post-metamorphic homeostasis. Treatment (fertilization to polyp; or planula to polyp) with the pan-caspase inhibitor Z-VAD does not affect metamorphosis (Fig.5A) but polyp size. Box plots indicating the body sizes obtained in the various treatments. N indicates the number of polyps used for the measurements. p-value (Z-VAD fertilization to polyp): 1.497078682128E-14; p-value (Z-VAD planula to polyp): 3.3426819863889E-7.

Figure S11

A

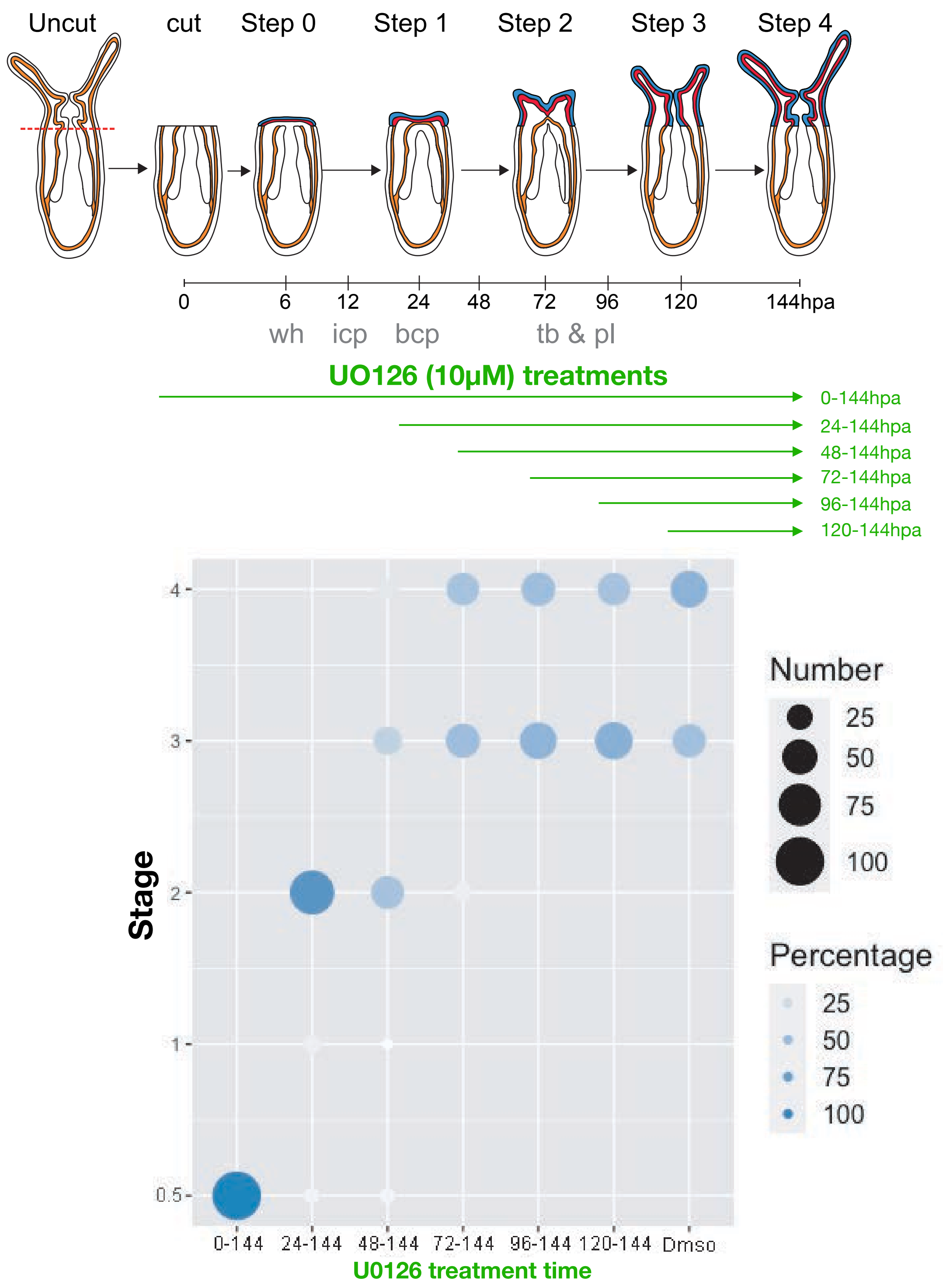

B

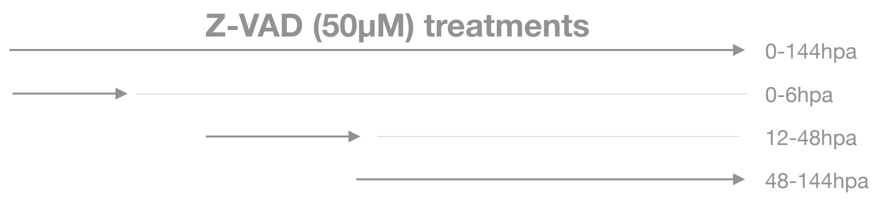

all 3 waves	0-144hpa fix 144hpa	*stage 0*	*%*	*stage 1*	*%*	*stage 2*	*%*	*stage 3*	*%*	*stage 4*	*%*	*N total*
	DMSO	0	0	2	3	3	4	11	16	53	77	**69**
	ZVAD	0	0	41*	76	5	9	0	0	8**	15	**54**
**1st wave**	**0-6hpa fix144hpa**											
	DMSO	0	0	0	0	0	0	12	17	57	83	**69**
	ZVAD	0	0	4	5	1	1	16	21	57**	73	**78**
**2nd wave**	**12-48hpa fix144hpa**											
	DMSO	0	0	0	0	0	0	21	24	68	76	**89**
	ZVAD	0	0	8*	11	6	9	19	27	37**	53	**70**
**3rd wave**	**48-144hpa fix 144hpa**											
	DMSO	0	0	1	2	1	2	19	32	38	64	**59**
	ZVAD	0	0	12*	18	6	9	14	21	36**	53	**68**

* 100% stage 1 modified
** 63% (5) with tentacle-less phenotype

* 0% stage 1 modified
** 21% (12) with tentacles less phenotype

* 13% stage 1 modified
** 32% (12) with tentacle-less phenotype

* 33% stage 1 modified
** 72% (26) with tentacle-less phenotype

Figure S11: U0126 or Z-VAD treatment waves. (A) Schematic representation of the various U0126 treatment periods that were applied during different windows of time during regeneration. Polyps were fixed at the end of each treatment and the regeneration phenotype assessed as indicated in the graph. (B) Schematic representation of the various Z-VAD treatment periods that were applied during different windows of time during regeneration. Inhibition periods spanned all three or only one of the described apoptosis waves activated upon injury. Polyps were fixed at the end of each treatment and the regeneration phenotype assessed as indicated in the table.

Figure S12

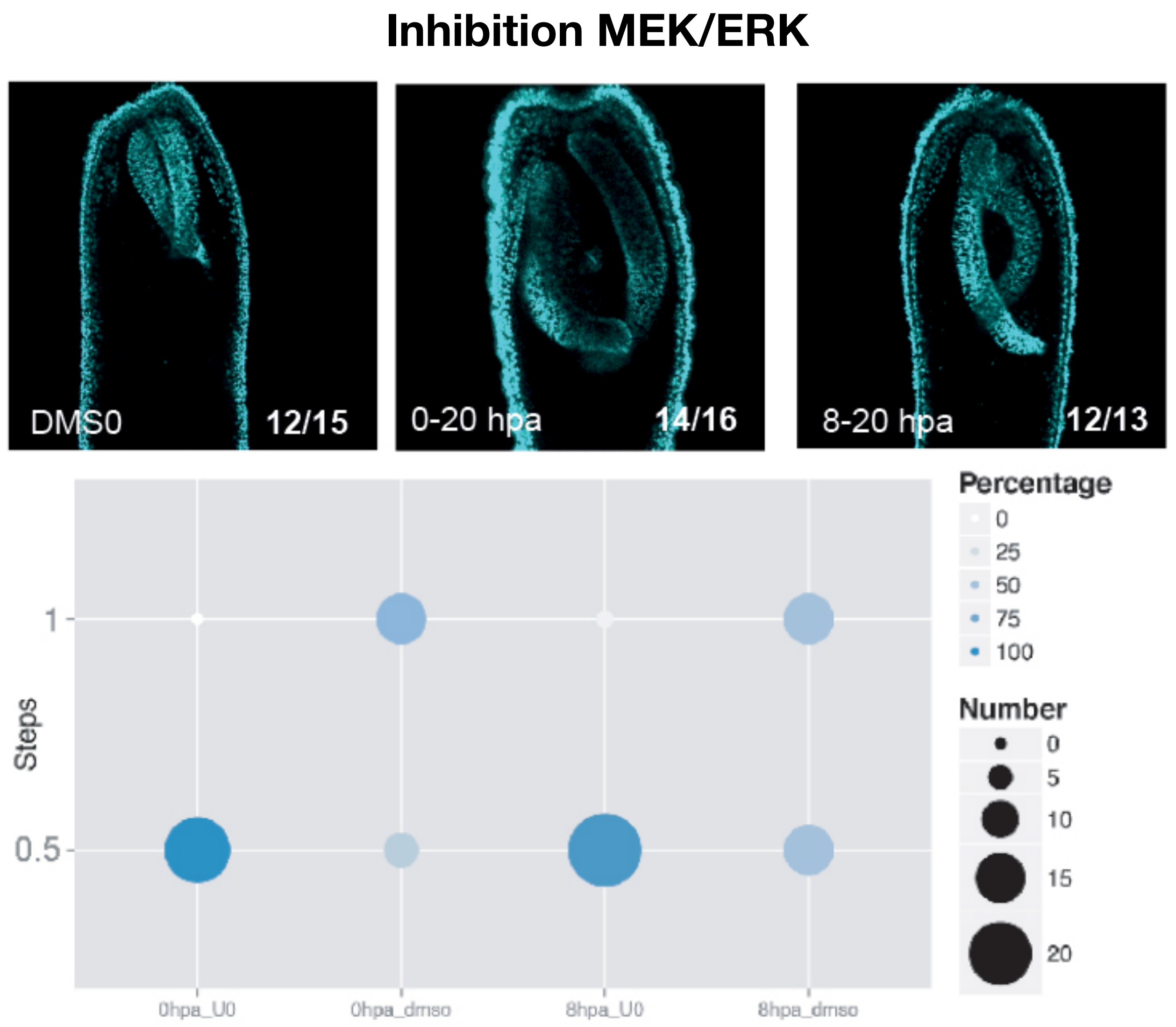

Figure S12: Inhibition of MEK/ERK between 8-20hpa phenocopies 0-20hpa treatments. Confocal stacks of regenerating polyps counterstained with DAPI (cyan) focused on the mesenteries at 20 hpa after a 0-20 or 8-20 hpa treatment with U0126. The dotplot shows the distribution of polyps between stages 0.5 and 1 according to the treatments.

Figure S13

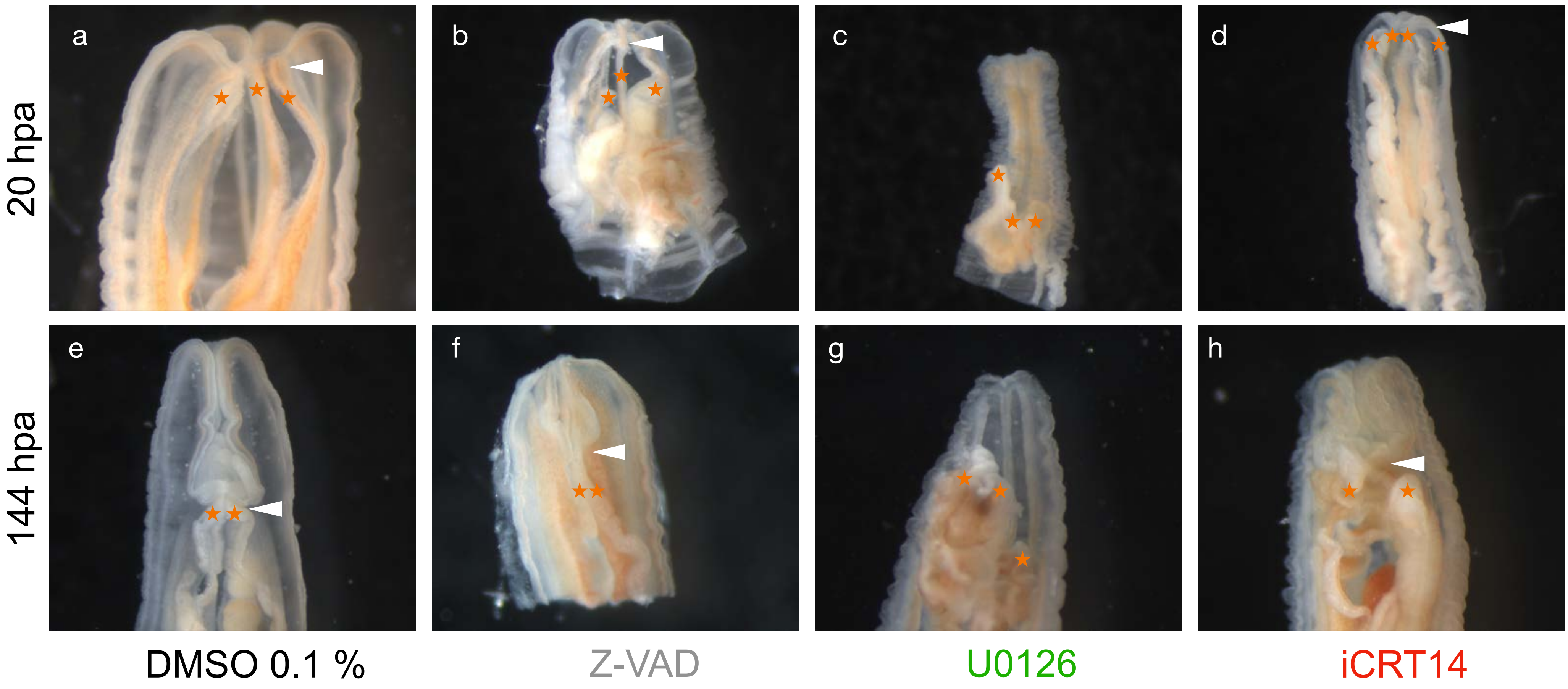

Figure S13: Z-VAD, U0126 or iCRT14 treatments cause the same phenotypes in adult polyps. Brightfield photographs on dissected polyps treated with DMSO (a,e), Z-VAD (b,f), U0126 (c,g) or iCRT14 (d,h) at 20 and 144hpa. White arrowheads indicate the TC, orange start the oral tip of the mesenteries.

Figure S14

DEG DMSO *vs* ZVAD

DOWN-regulated genes

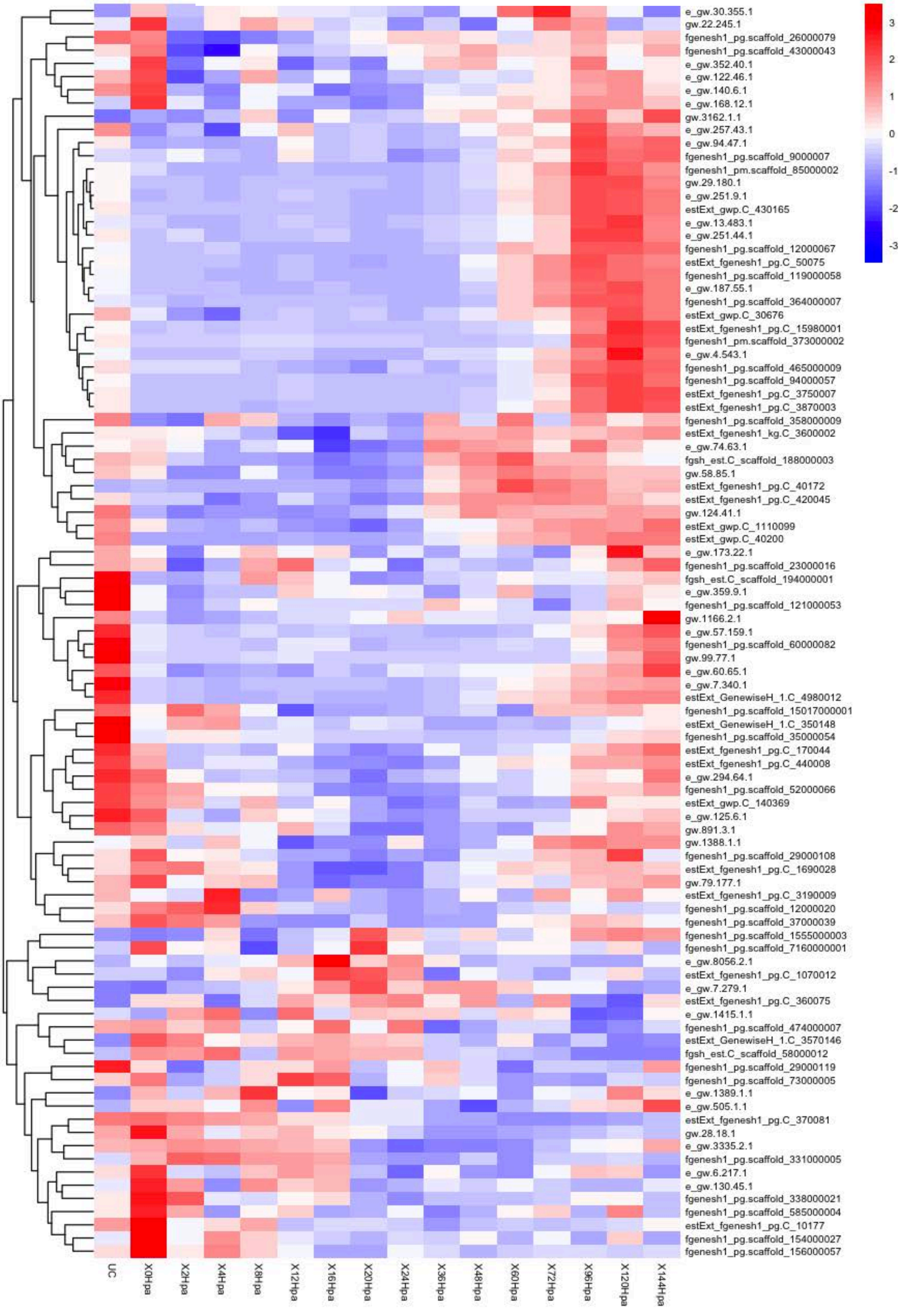

DEG DMSO *vs* **ZVAD**

GO terms DOWN-regulated genes

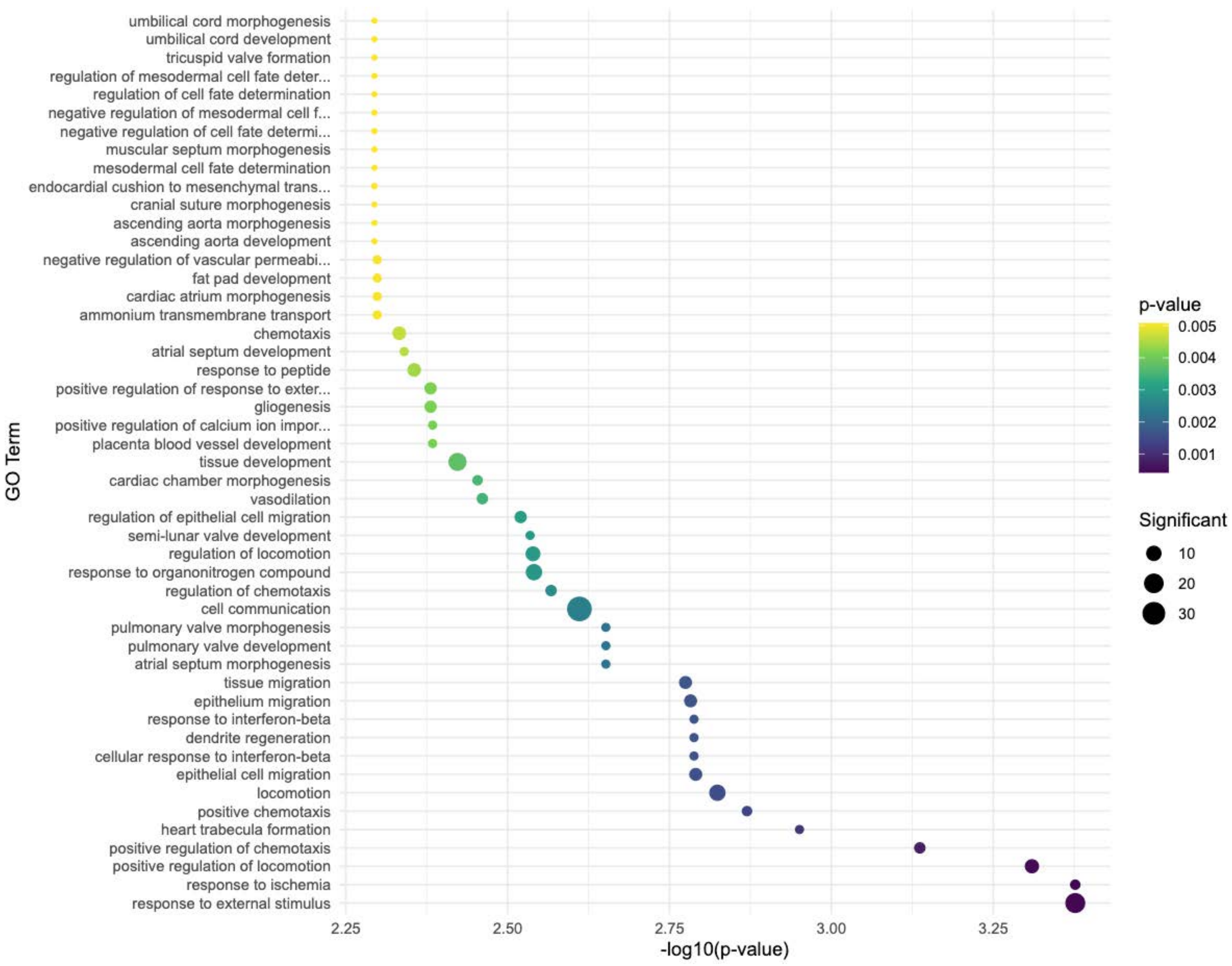

DEG DMSO *vs* **ZVAD**

UP-regulated genes

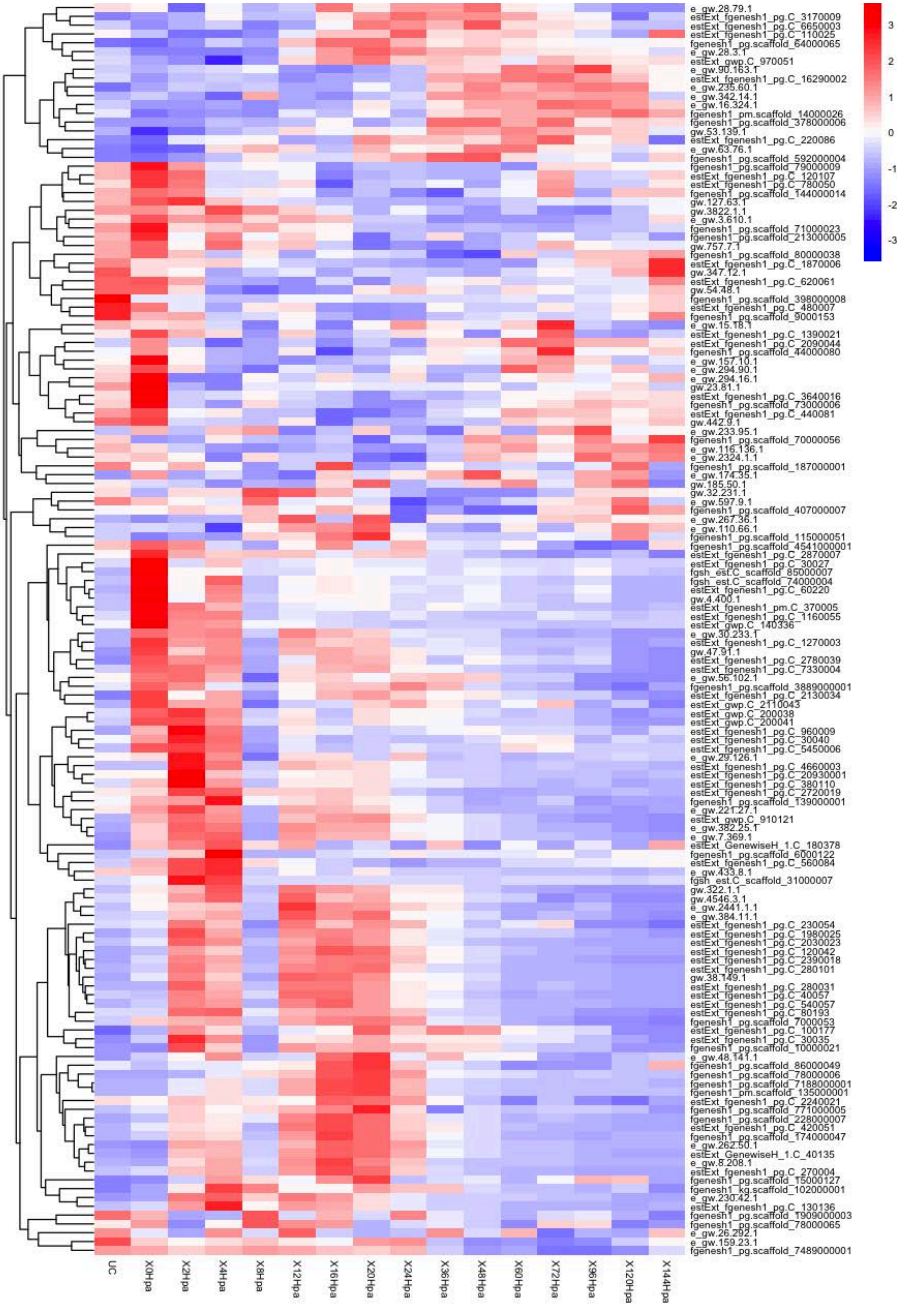

DEG DMSO *vs* **ZVAD**

GO terms UP-regulated genes

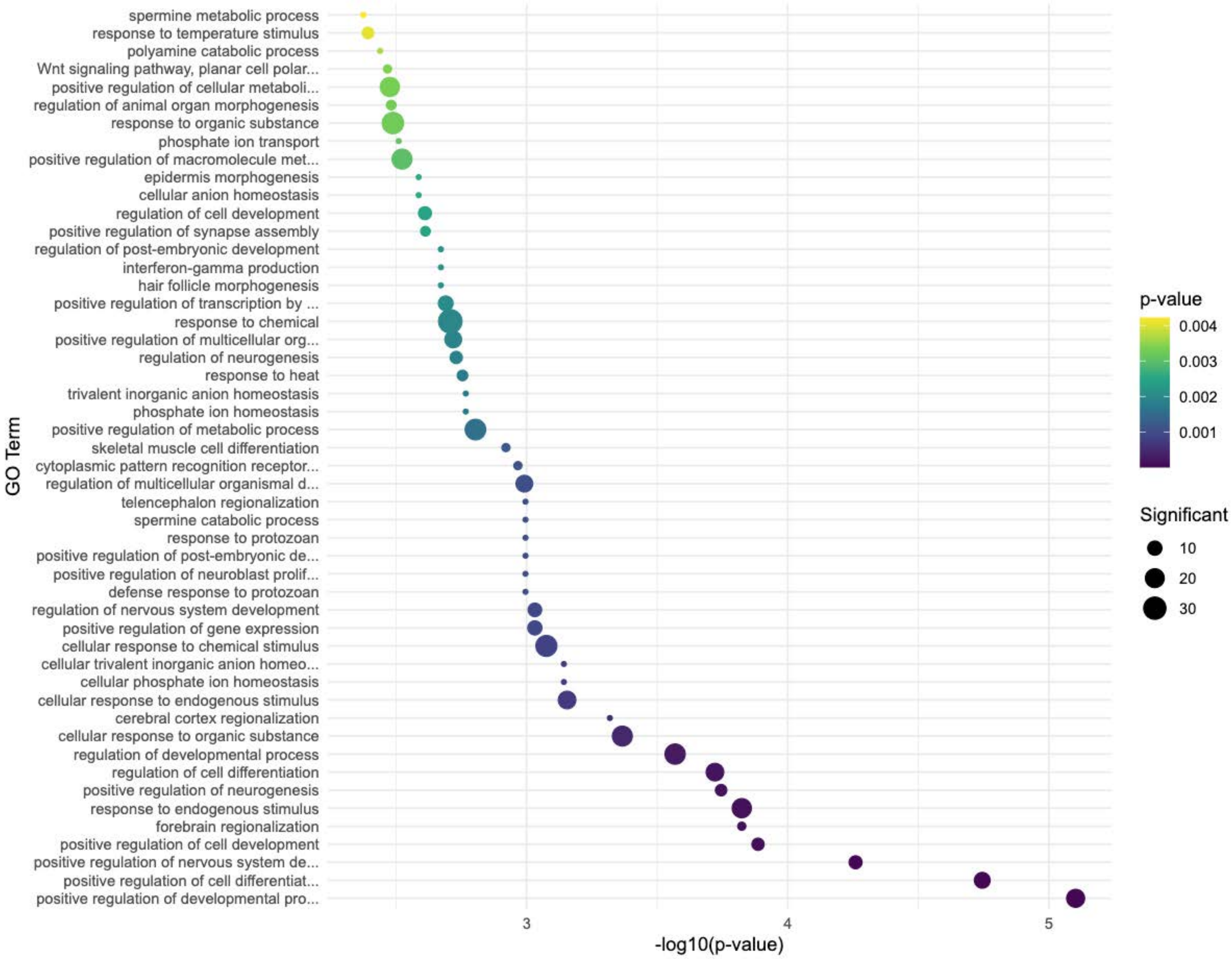

DEG DMSO *vs* **U0126**

DOWN-regulated genes

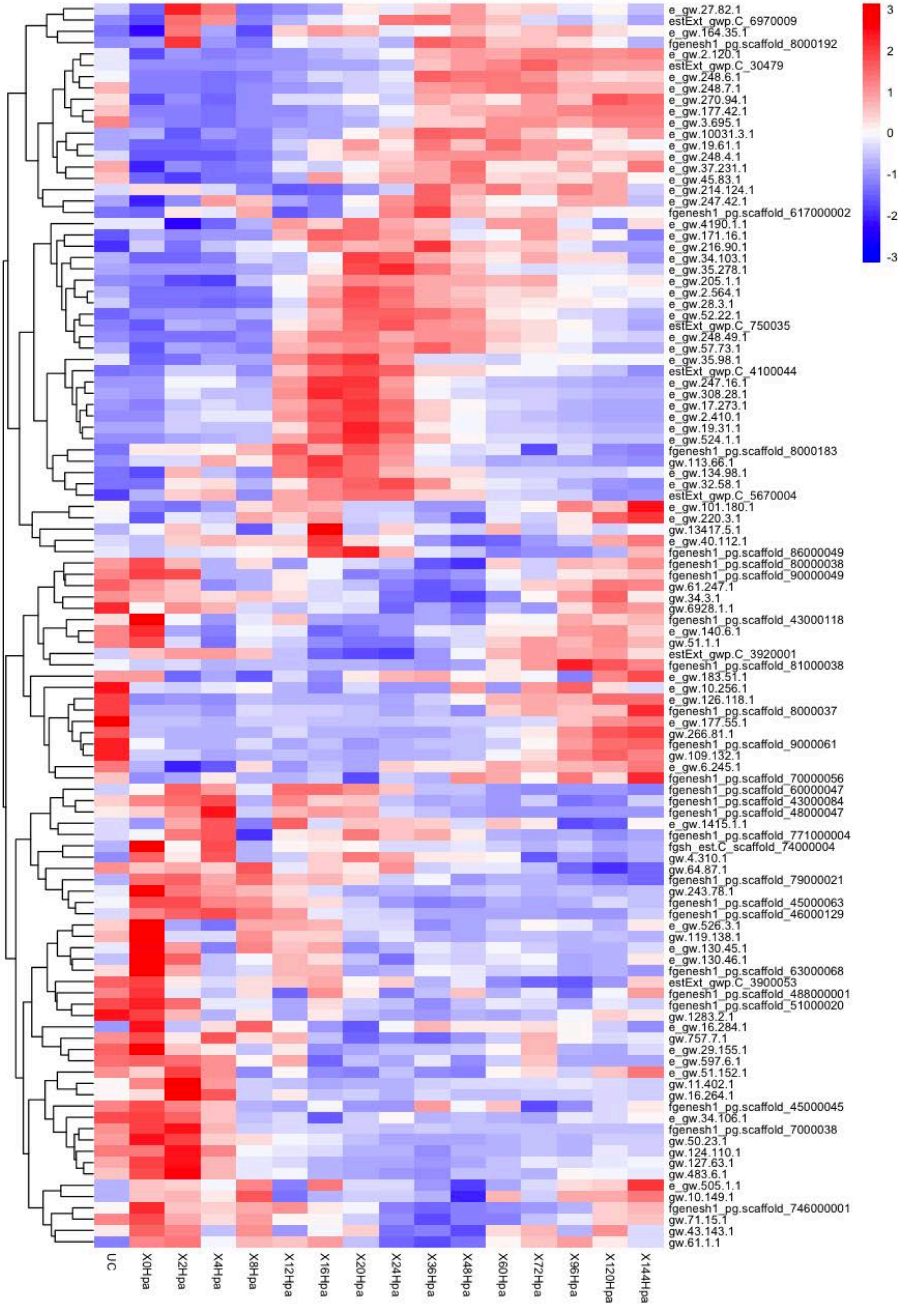

DEG DMSO *vs* **U0126**

GO terms DOWN-regulated genes

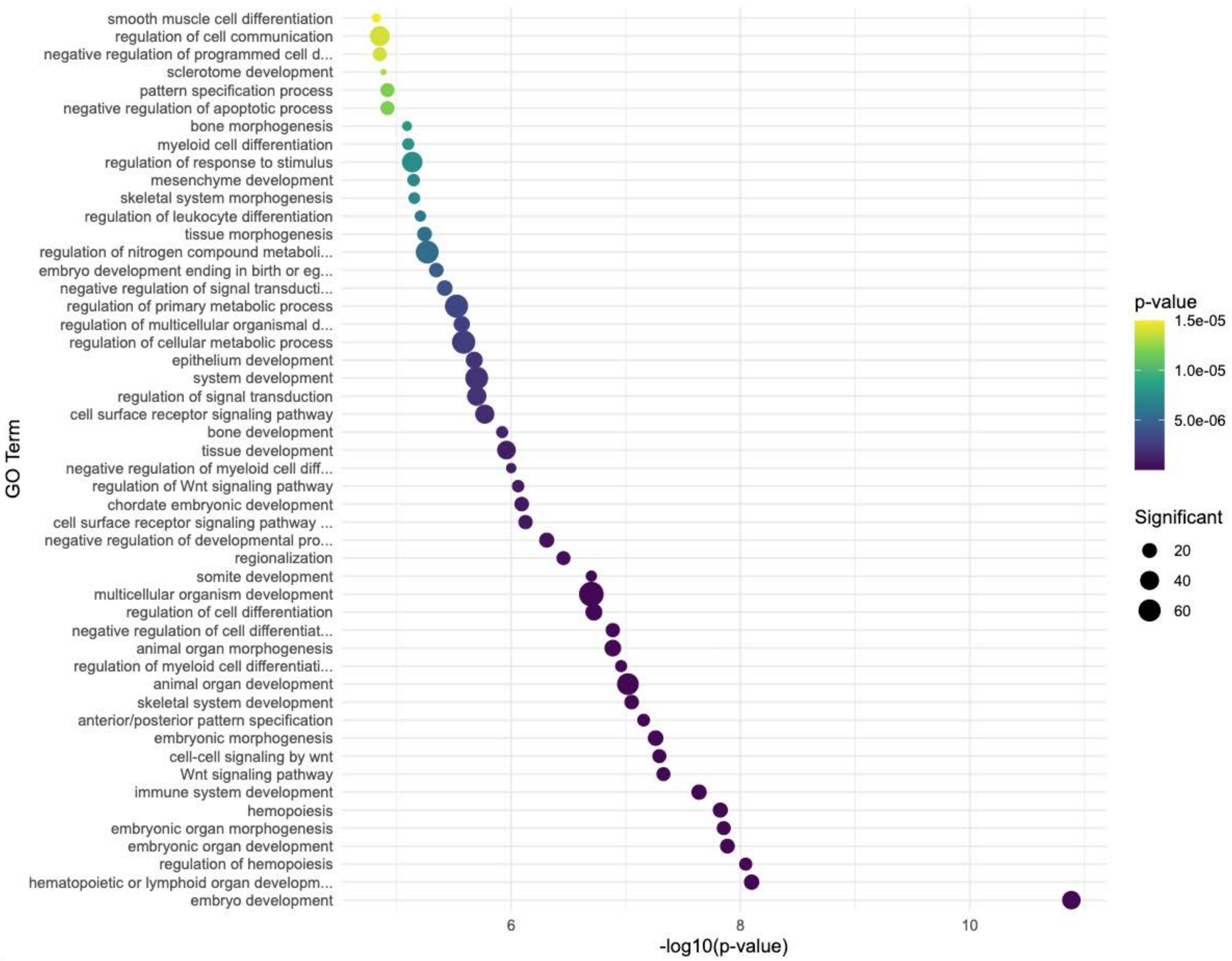

DEG DMSO *vs* **U0126**

UP-regulated genes

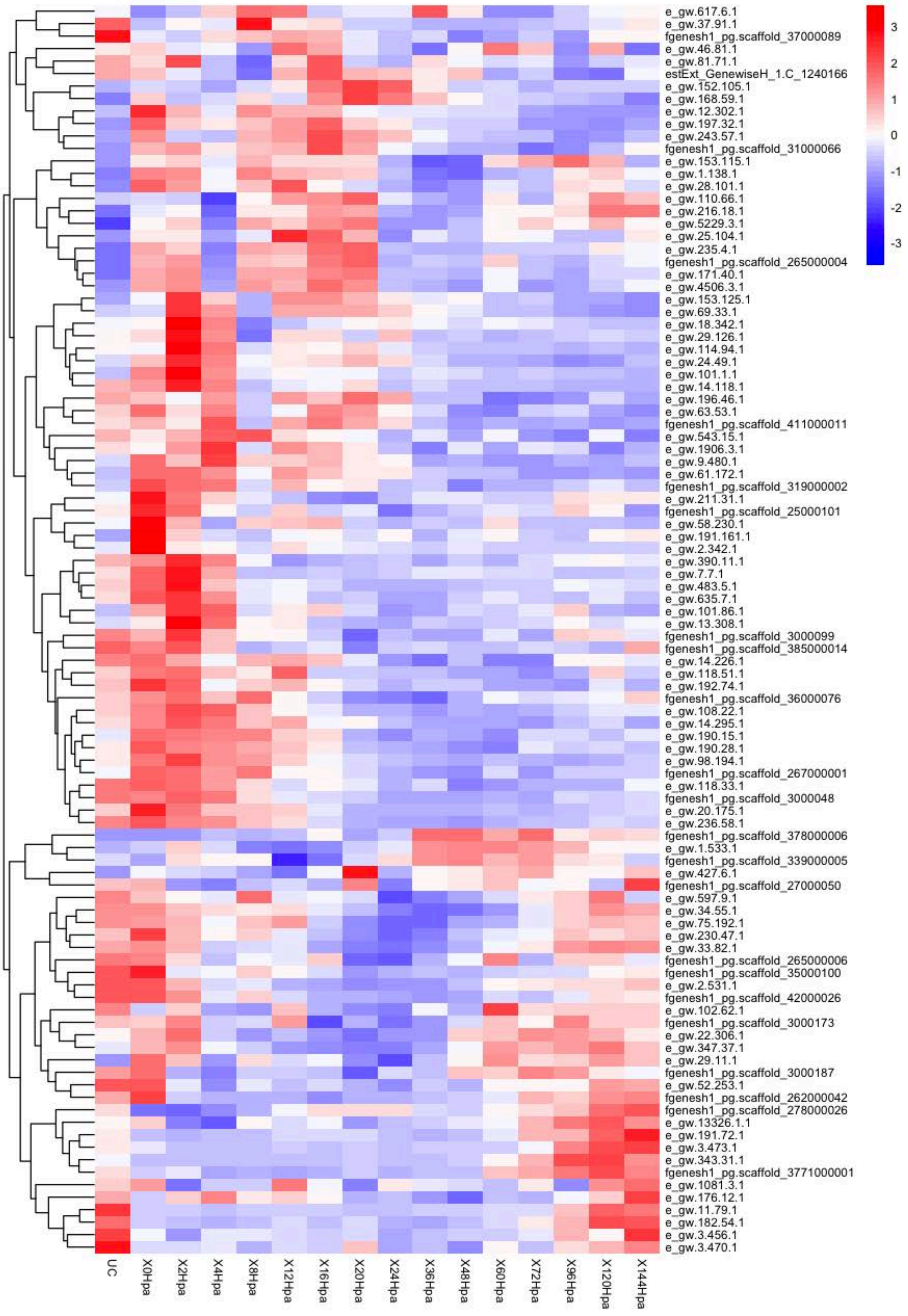

DEG DMSO *vs* **U0126**

GO terms UP-regulated genes

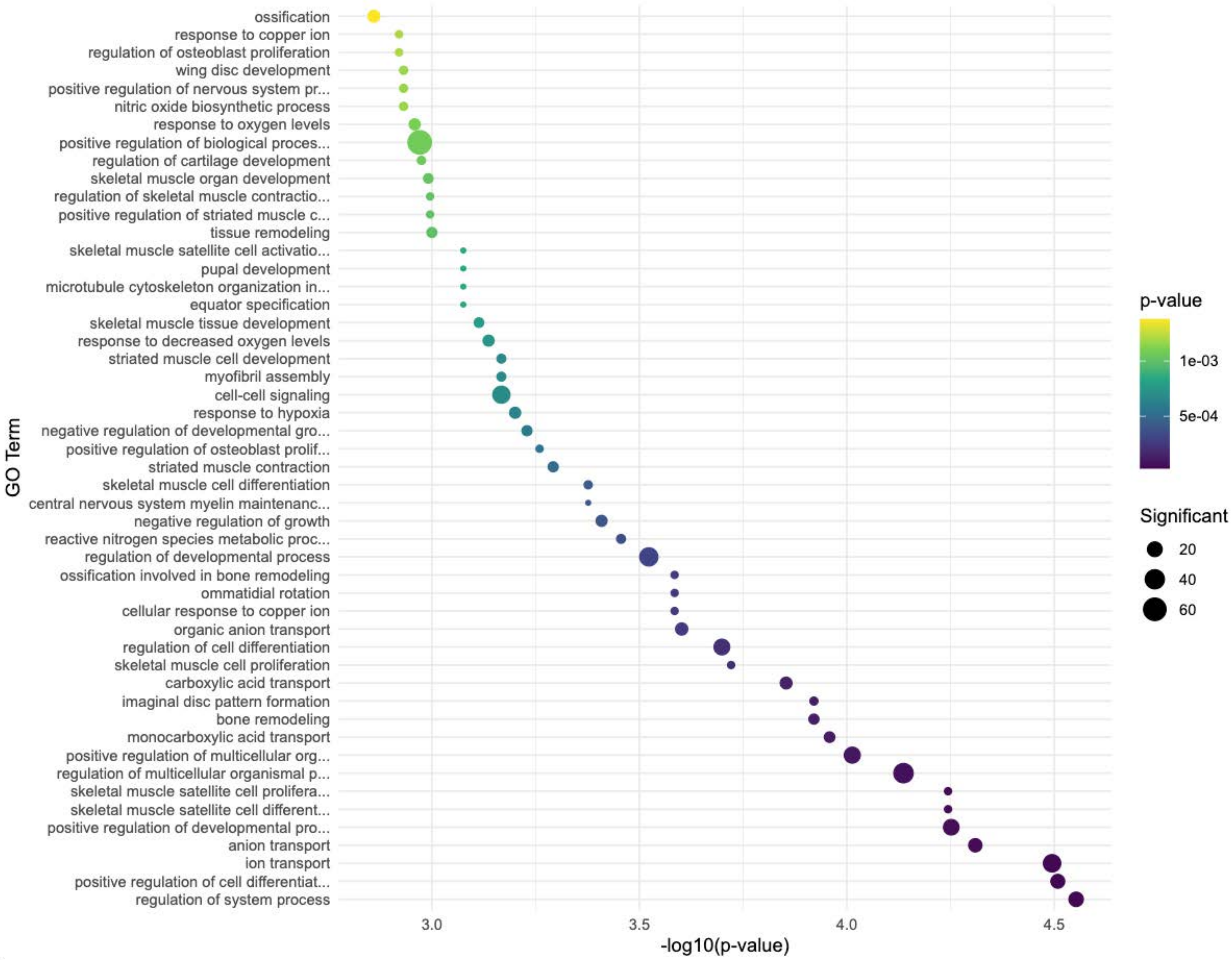

DEG DMSO *vs* iCRT14

DOWN-regulated genes

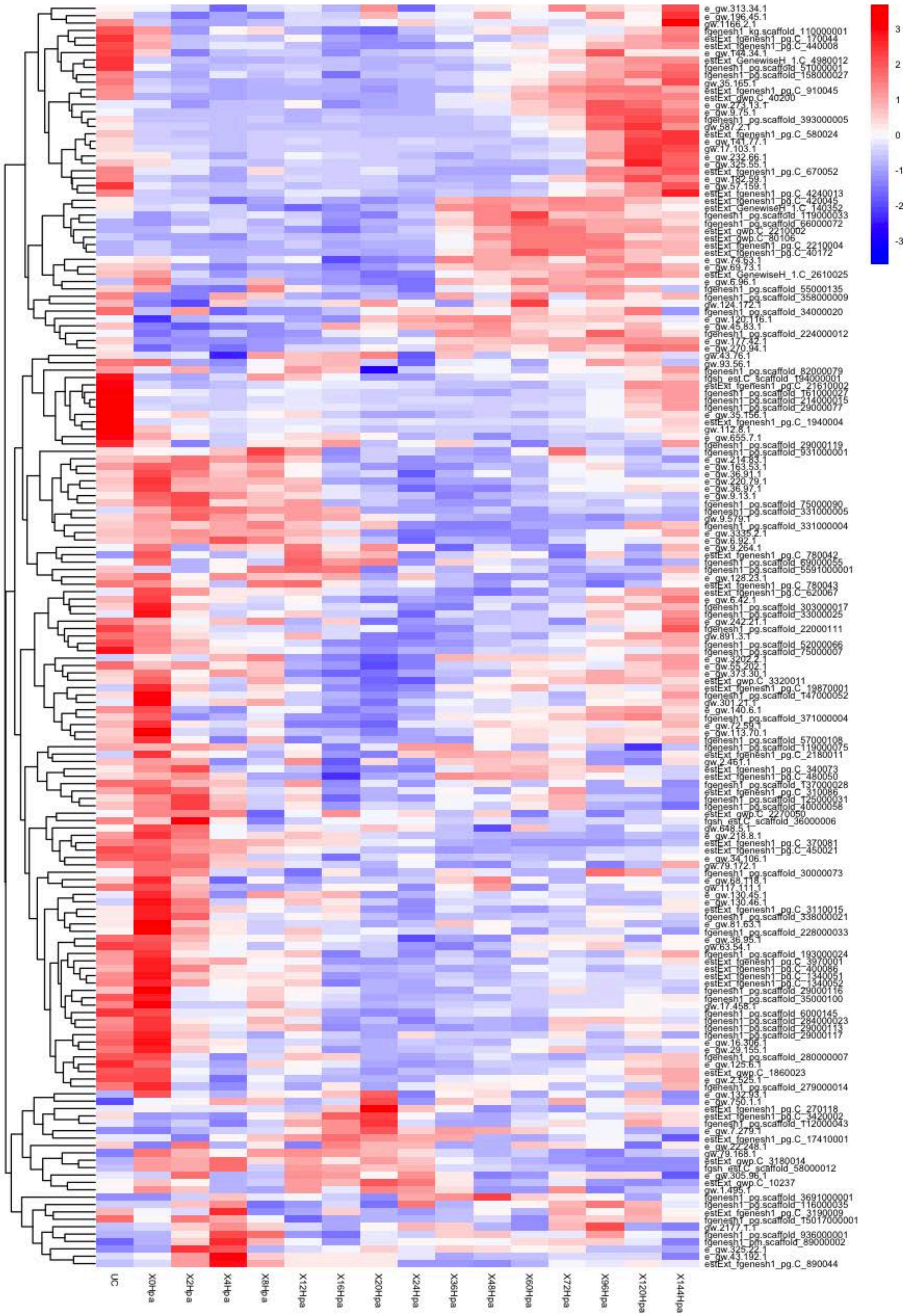

DEG DMSO *vs* **iCRT14**

GO terms DOWN-regulated genes

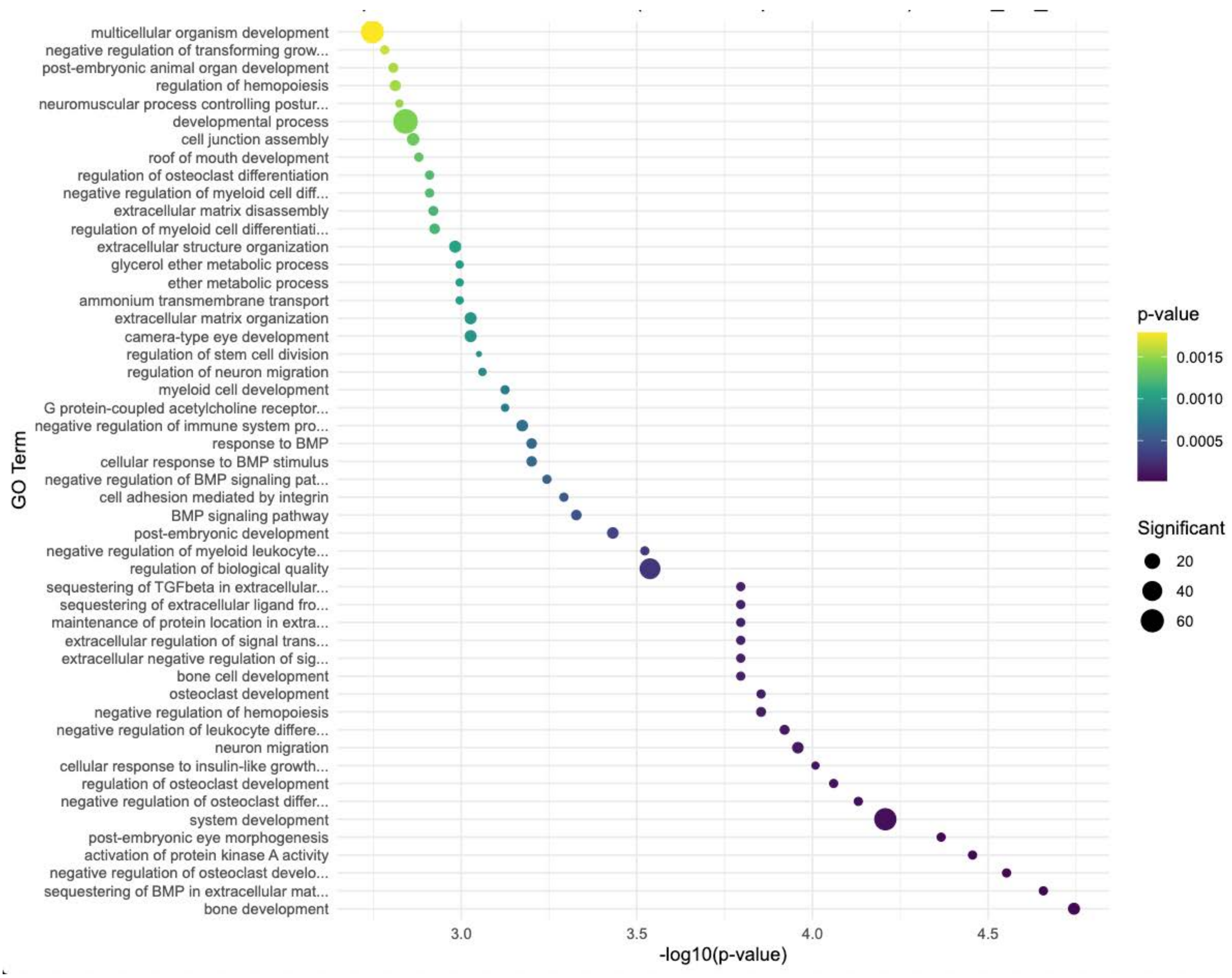

DEG DMSO *vs* iCRT14

UP-regulated genes

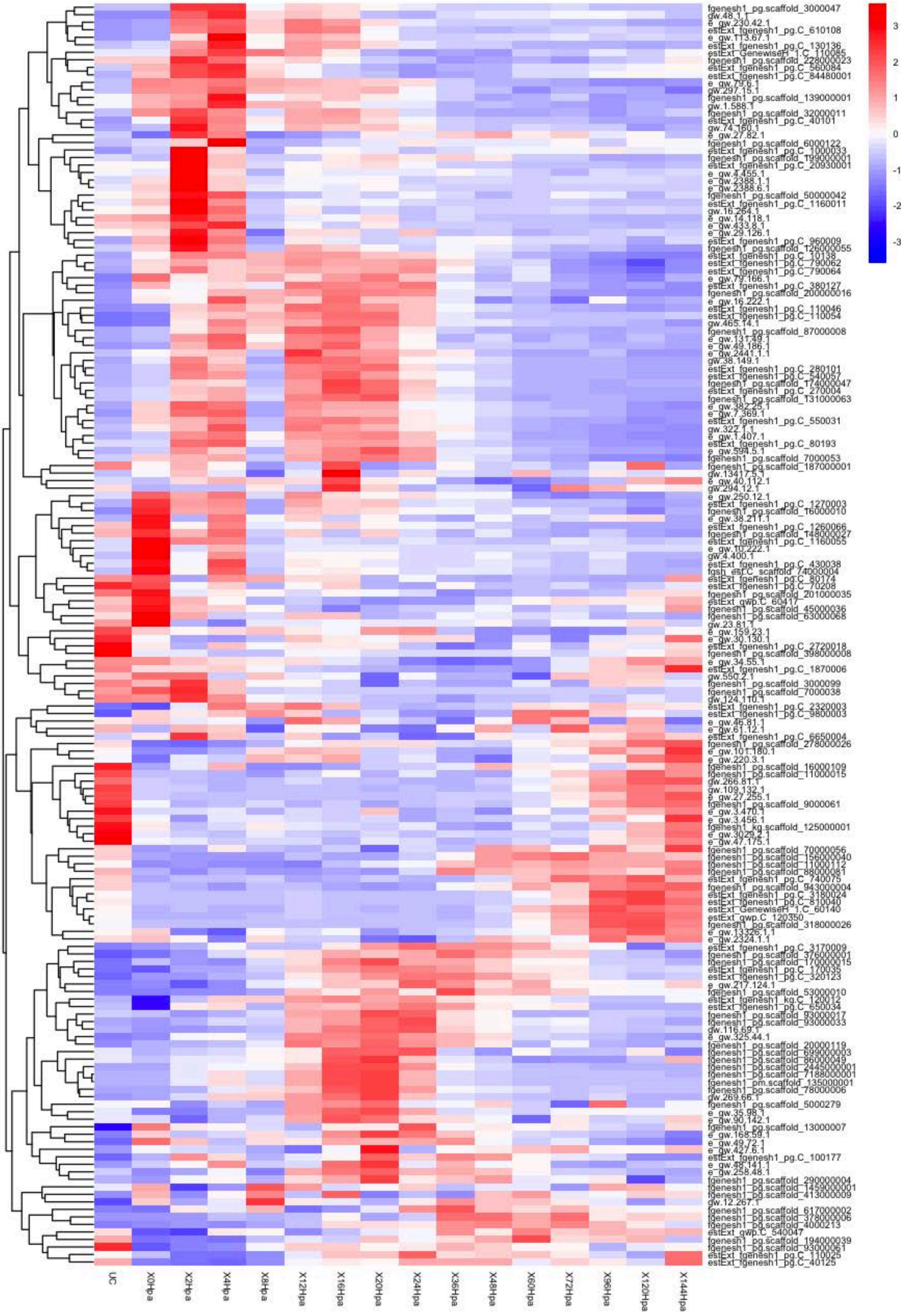

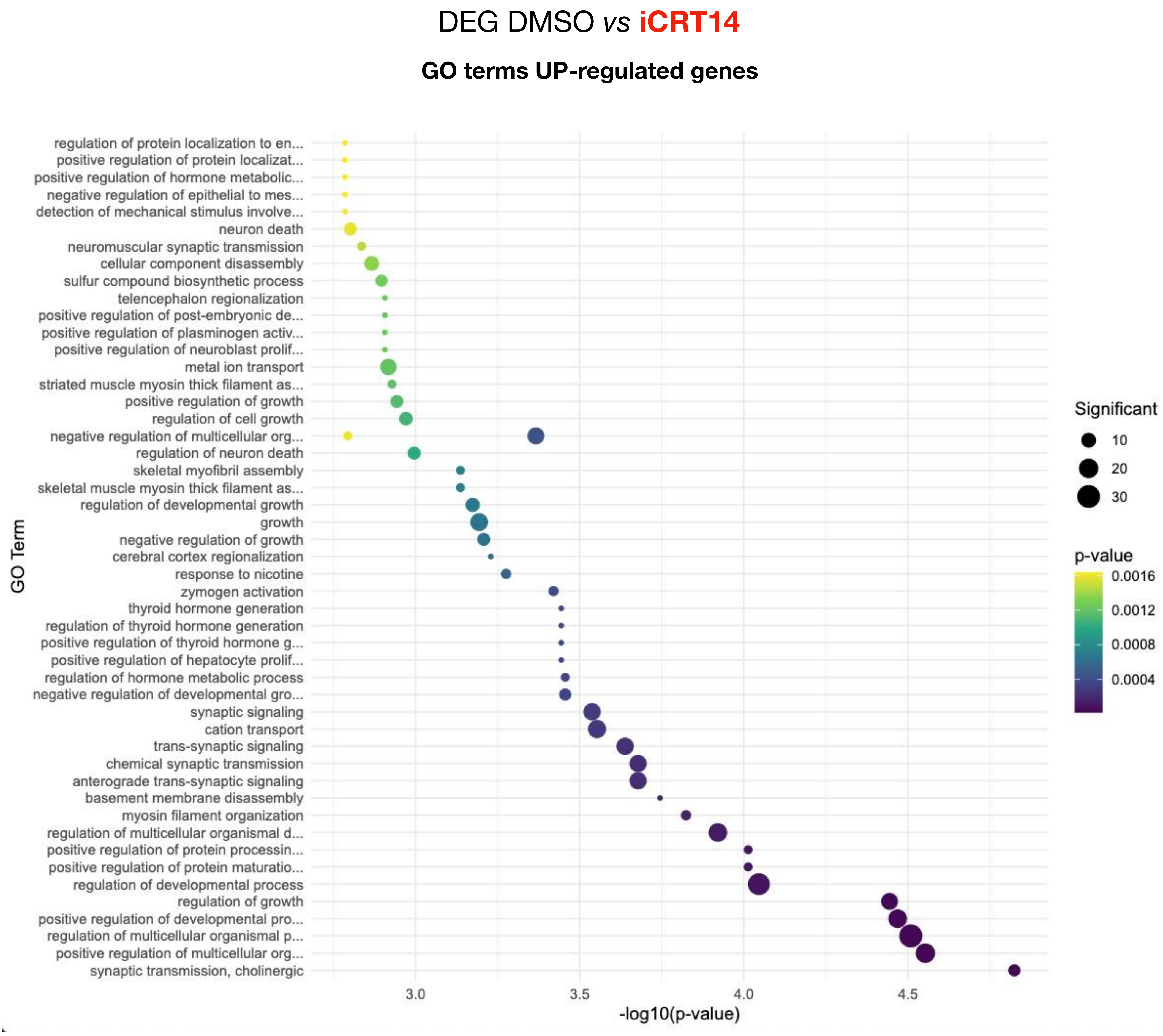

Figure S14: Heatmaps indicating temporal gene expression during regeneration of the up- or down-regulated DEGs following Z-VAD, U0126 or iCRT14 treatments & associated GO term enrichment plots.

The heatmaps were generated using the R package pheatmap with log2-transformed expression values. Rows represent individual genes, and columns correspond to temporal sampling points. Red indicates higher expression levels, and blue indicates lower expression levels. Hierarchical clustering was applied to genes to highlight similar expression patterns across time. Top 50 Go-term enrichment plots for the genes down- or up-regulated by Z-VAD, U0126 or iCRT14 at 20hpa. More details can be found in Supplementary table 17.

Figure S15

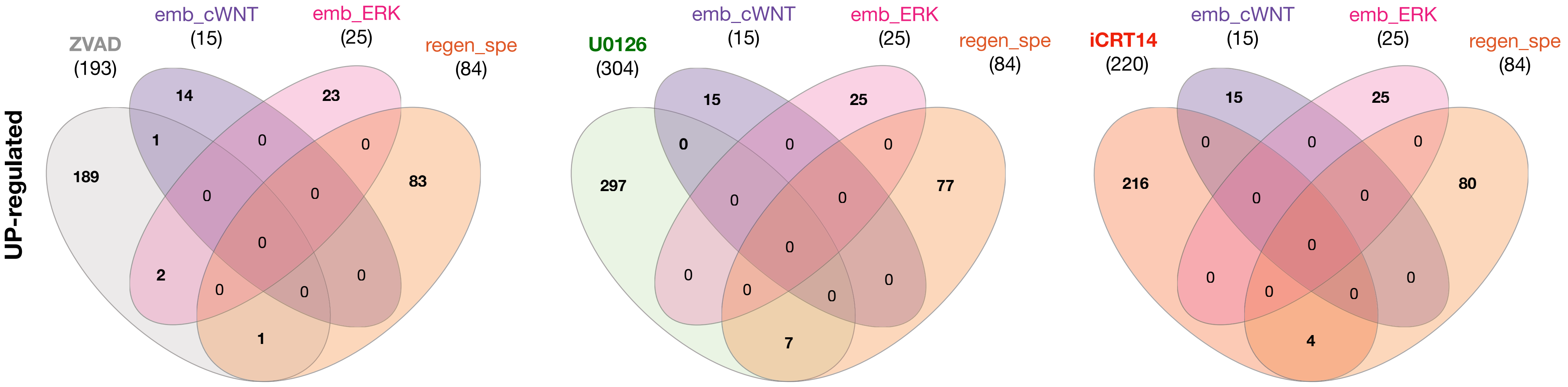

Figure S15: MEK/ERK controls embryonic cWnt downstream targets as well as regeneration-specific genes during regeneration. Venn diagrams illustrating the comparison of the up-regulated differentially expressed genes (DEGs) from either Z-VAD, U0126 or iCRT14 with the emb_cWnt, emb_ERK or regeneration-specific (regen_spe) datasets. Venn diagrams illustrating the corresponding down-regulated DEGs can be found in Figure 8A.

Figure S16

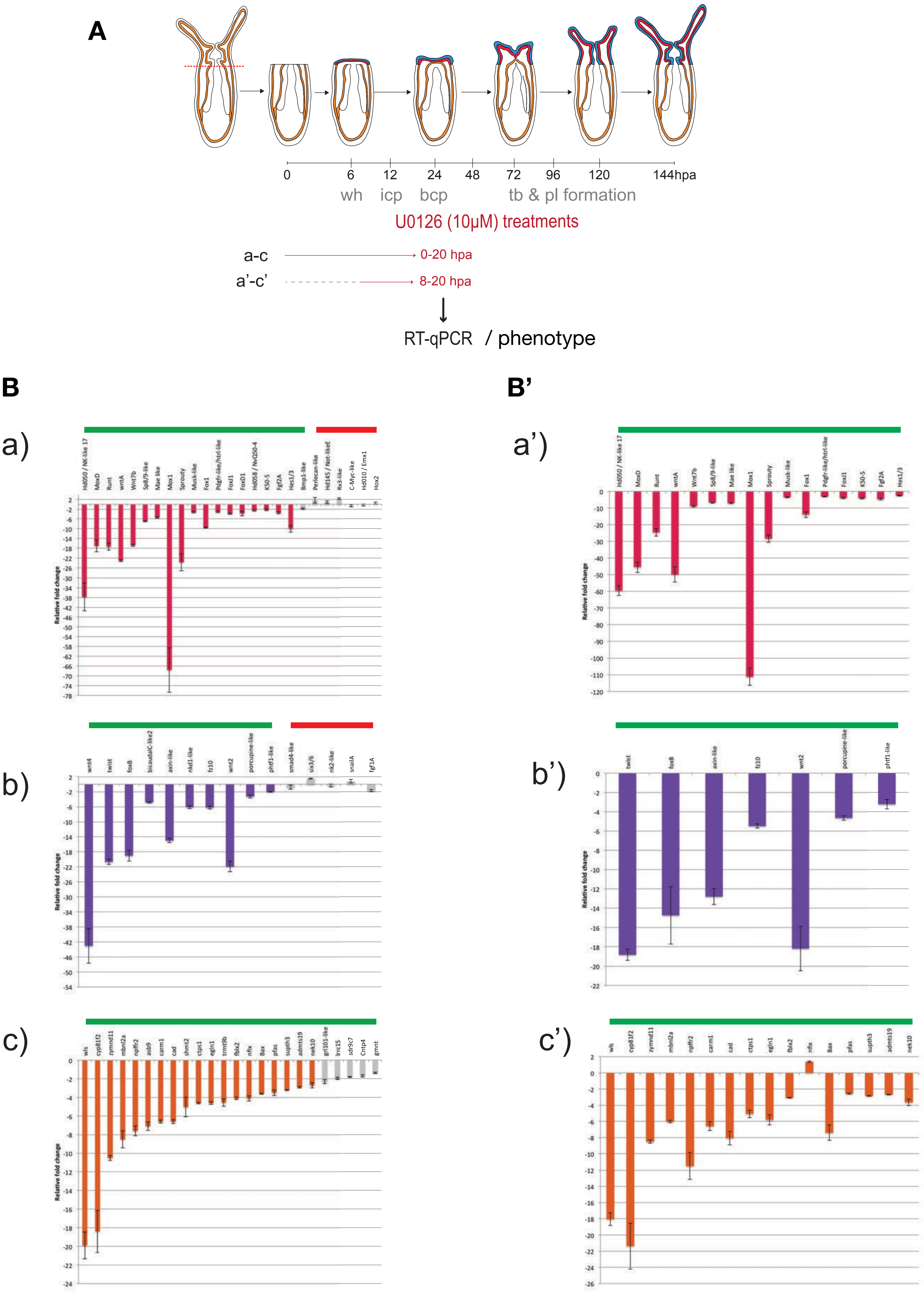

Figure S16: Inhibition of MEK/ERK between 8-20hpa or 0-20hpa treatments have a similar molecular effect. (A) Diagram of the experimental set-up. (wh) wound healing, (icp) induction of cell proliferation, (bcp) burst of cell proliferation (tb) tentacle buds and (ph) pharynx formation. (B,B') Comparison of the RT-qPCR analysis on regenerating polyps treated from 0-20hpa (Ba-c) or from 8-20hpa (B'a'-c') with the same primer sets indicating that both treatment periods yield comparable results.

Figure S17

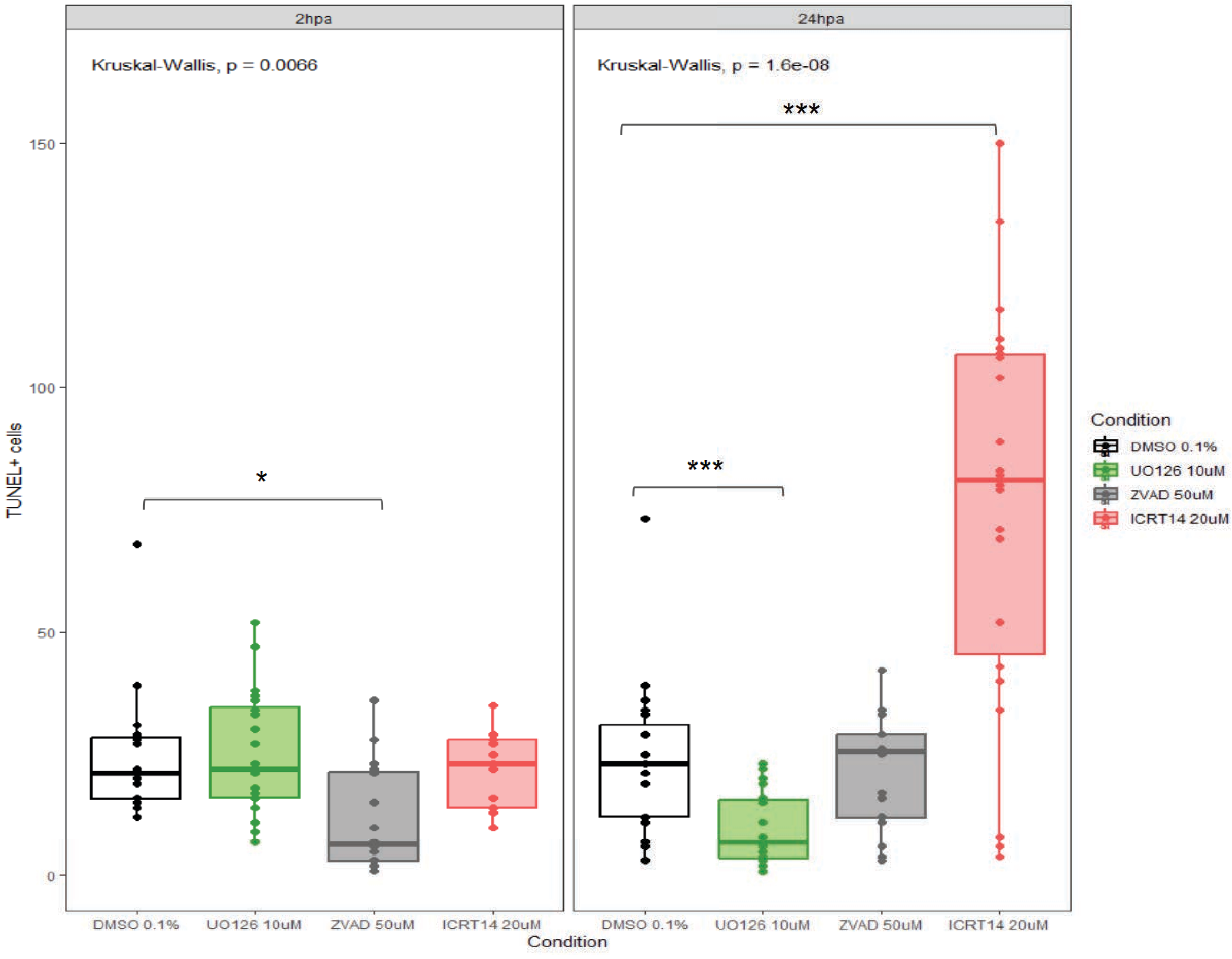

Figure S17: TUNEL+ cell dynamics following inhibitory treatments at 2hpa and 20hpa. Dot-plots showing the distribution of TUNEL+ cells for each sample under different inhibitory treatments. Statistical significance was assessed using Wilcoxon paired tests comparing each treatment to DMSO for each time-point. Significance is indicated as follows: p < 0.05 (), p < 0.001 (**), indicating moderate or high significant differences, respectively.
