# Supplementary material for: Whole body regeneration deploys a rewired embryonic gene regulatory network logic
Source: Nat Commun. 2025 Dec 11;17:503. doi: 10.1038/s41467-025-67196-4 (PMC12804838; doi:10.1038/s41467-025-67196-4)
Supplement: Supplementary file 2 — Description of Additional Supplementary Files [file 41467_2025_67196_MOESM2_ESM.pdf]

Description of Additional Supplementary Files

Supplementary Data 1: “123 Regeneration-specific” gene list

Supplementary Data 2: GO Term enrichment – embryonic development. GO-term enrichment was assessed using a one-sided Fisher’s exact test ($P < 0.02$).

Supplementary Data 3: GO Term enrichment – regeneration. GO-term enrichment was assessed using a one-sided Fisher’s exact test ($P < 0.02$).

Supplementary Data 4: Differential Gene Expressions (DEGs) at 20hpa (vs. 0hpa).

Supplementary Data 5: Embryonic cWnt downstream targets

Supplementary Data 6: Embryonic ERK downstream targets

Supplementary Data 7: 20hpa - Embryonic ERK downstream targets

Supplementary Data 8: 20hpa - Embryonic cWnt downstream targets

Supplementary Data 9: 20hpa - regeneration specific genes

Supplementary Data 10: Tissue contact stages

Supplementary Data 11: EdU + cell counting

Supplementary Data 12: Raw count bulk-RNAseq under treatments

Supplementary Data 13: Adult treatments at 20hpa

Supplementary Data 14: 20hpa U0126 DEGs

Supplementary Data 15: 20hpa Z-VAD DEGs

Supplementary Data 16: 20hpa iCRT14 DEGs

Supplementary Data 17: GO Term DEGs treatments. GO-term enrichment was assessed using a one-sided Fisher’s exact test ($P < 0.02$).

Supplementary Data 18: Down-regulated DEGs all 3 pathways

Supplementary Data 19: Up-regulated DEGs all 3 pathways

Supplementary Data 20: Down-regulated DEGs in U0126 compared to embryonic GRN and regeneration-specific genes

Supplementary Data 21: Up-regulated DEGs in U0126 compared to embryonic GRN and regeneration-specific genes

Supplementary Data 22: Down-regulated DEGs in Z-VAD compared to embryonic GRN and regeneration-specific genes

Supplementary Data 23: Up-regulated DEGs in Z-VAD compared to embryonic GRN and regeneration-specific genes

Supplementary Data 24: Down-regulated DEGs in iCRT14 compared to embryonic GRN and regeneration-specific genes

Supplementary Data 25: Up-regulated DEGs in iCRT14 compared to embryonic GRN and regeneration-specific genes

Supplementary Data 26: Statistic analysis. p-value for the Wound healing analysis (two sided Fisher exact test with BH correction), Edu Counting (two sided Wilcoxon–Mann–Whitney tests with BH correction (non-parametric)), for Luciferase assay (two sided Kruskal Wallis test, two sided Dunn test with BH correction).

Supplementary Movie 1: Timelapse movie of the Nvß-catenin::mCherry embryo (0-8hpf), indicating the nuclearization of Nvß-catenin during cleavage stages.
